# A reciprocal interaction between L‐lysine and *Holdemanella biformis* modulates intestinal barrier function and anxiety in irritable bowel syndrome

**DOI:** 10.1002/imo2.70042

**Published:** 2025-08-06

**Authors:** Chun‐Hui Jiang, Xue Fang, Wen Huang, Ya‐Hui Wang, Le Kang, Peng‐Yuan Wang, Chao Xu, Zhao‐Shen Li, Wen‐Bin Zou, Zhuan Liao

**Affiliations:** ^1^ Department of Gastroenterology, Shanghai Key Laboratory of Nautical Medicine and Translation of Drugs and Medical Devices, Changhai Hospital; National Key Laboratory of Immunity and Inflammation Naval Medical University Shanghai China

**Keywords:** anxiety, *Holdemanella biformis*, intestinal barrier function, irritable bowel syndrome, l‐lysine, tryptophan metabolism pathway

## Abstract

Irritable bowel syndrome (IBS) is a prevalent stress‐associated disorder characterised by gut barrier dysfunction and gut‐brain axis disturbances. However, the interplay between host metabolites and gut microbes in IBS pathogenesis remains incompletely understood. Here, through integrated microbiome and metabolome profiling of faecal sample from seafarers before and after long‐term voyages, we identify a reciprocal interaction between the essential amino acid l‐lysine and the gut bacterium *Holdemanella biformis* (*H. biformis*). l‐lysine was depleted in individuals with voyage‐induced IBS, whereas *H. biformis* abundance increased concurrently. In a mouse model of diarrhoea‐predominant IBS, l‐lysine supplementation restored intestinal barrier integrity, reduced visceral hypersensitivity, and alleviated anxiety‐like behaviours through modulation of tryptophan metabolism. In contrast, oral administration of *H. biformis* improved tight junction protein expression but paradoxically worsened anxiety‐like phenotypes. In vitro, both l‐lysine and *H. biformis* supernatant promoted epithelial wound healing and ZO‐1 expression. Mechanistically, *H. biformis* degrades l‐lysine via lysine degradation pathway, while l‐lysine suppresses *H. biformis* growth possibly by downregulating pathways involved in carbohydrate and energy metabolism. These findings reveal a stress‐sensitive, bidirectional metabolic loop in between l‐lysine and *H. biformis*, with complementary but opposing effects on gut and neurological function. Targeting this axis may offer new strategies for IBS and other gut‐brain axis disorders.

## Highlights

- l‐lysine improves intestinal barrier function and alleviates anxiety‐like behaviours by modulating the tryptophan metabolism pathway.
- *Holdemanella biformis* (*H. biformis*) enhances intestinal barrier function but exacerbates anxiety‐like behaviours.
- *H. biformis* metabolises l‐lysine primarily by the lysine degradation pathway, while l‐lysine inhibits *H. biformis* growth, likely through downregulation of carbohydrate and energy metabolism pathways.

## Graphical abstract

This study uncovers a complex interaction between l‐lysine and *Holdemanella biformis* (*H. biformis*) in irritable bowel syndrome (IBS). l‐lysine enhances intestinal barrier function and alleviates anxiety‐like behaviours by modulating tryptophan metabolism. In contrast, *H. biformis* improves gut barrier integrity but exacerbates anxiety‐like behaviours. *H. biformis* degrades l‐lysine, whereas l‐lysine inhibits *H. biformis* growth. These findings provide new insights into microbiota‐metabolite interactions in IBS and suggest that target modulation of the l‐lysine‐*H. biformis* axis may offer novel therapeutic strategies for IBS and gut‐brain axis disorders.

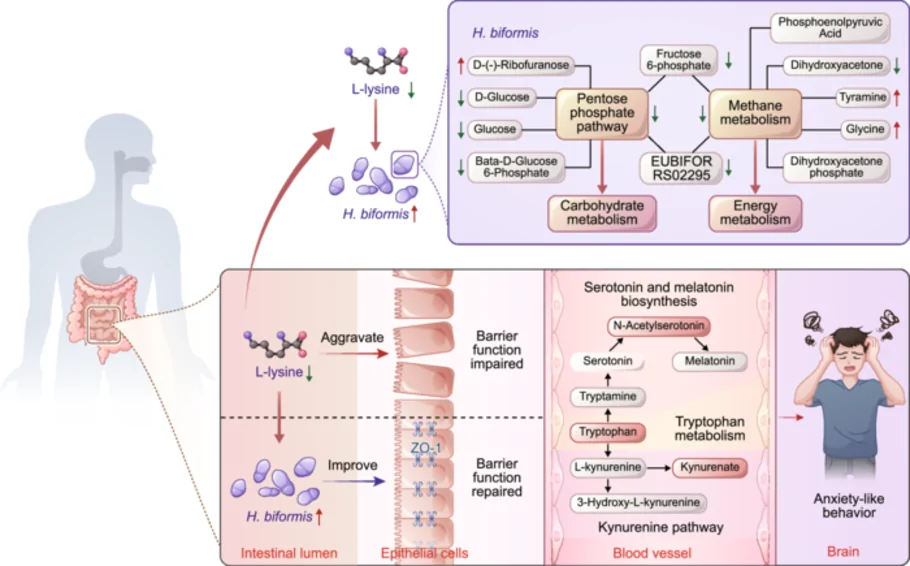

## 1 INTRODUCTION

Irritable bowel syndrome (IBS) is a common gastrointestinal disease characterised by dysfunction of the brain–gut axis and is closely associated with disturbances in the intestinal microecosystem [1, 2]. Emerging evidence indicates that the chronic stressors encountered during long‐term voyages, including limited food supplies, harsh maritime conditions, circadian rhythm disorder, and turbulence, can induce intestinal dysbiosis and led to voyage‐associated functional gastrointestinal disorders, with IBS being the most prevalent [3, 4, 5]. Previously, we described the gut microbiome and metabolome landscape of seafarers before and after extended voyages [6]. Building on these data, we performed further omics analyses and intestinal disease enrichment of key differentially abundant microbes and metabolites, uncovering a potential link between l‐lysine, *Holdemanella biformis* (*H. biformis*), and IBS.

l‐lysine is an essential amino acid critical for maintaining intestinal integrity and regulating mood [7, 8]. It has been shown to alleviate IBS symptoms and anxiety by modulating serotonin release in the central nucleus of the amygdala and acting as a 5‐hydroxytryptamine receptor 4 (5‐HT_4_) antagonist in the intestine [8, 9]. *H. biformis* is a commensal anaerobe of the human gut microbiota within the family *Erysipelotrichaceae* [10], and its abundance is reportedly reduced in IBS patients [11, 12]. Notably, our preliminary multi‐omics data revealed a negative correlation between l‐lysine levels and *H. biformis* abundance in seafarers with long‐term voyage‐induced IBS symptoms, suggesting a potential regulatory interplay [6].

In this study, we investigated the interaction between l‐lysine and *H. biformis*, and their roles in modulating intestinal barrier function and anxiety‐like behaviours in IBS. These findings offer new insights into the microbe‐metabolite interactions underlying IBS and inform the development of microbiota‐based therapeutic strategies, particularly in stress‐related conditions such as long‐term maritime exposure.

## 2 RESULTS

### Associations between l‐lysine, *H. biformis,* and IBS revealed by integrated multi‐omics and database analysis

**FIGURE 1:**
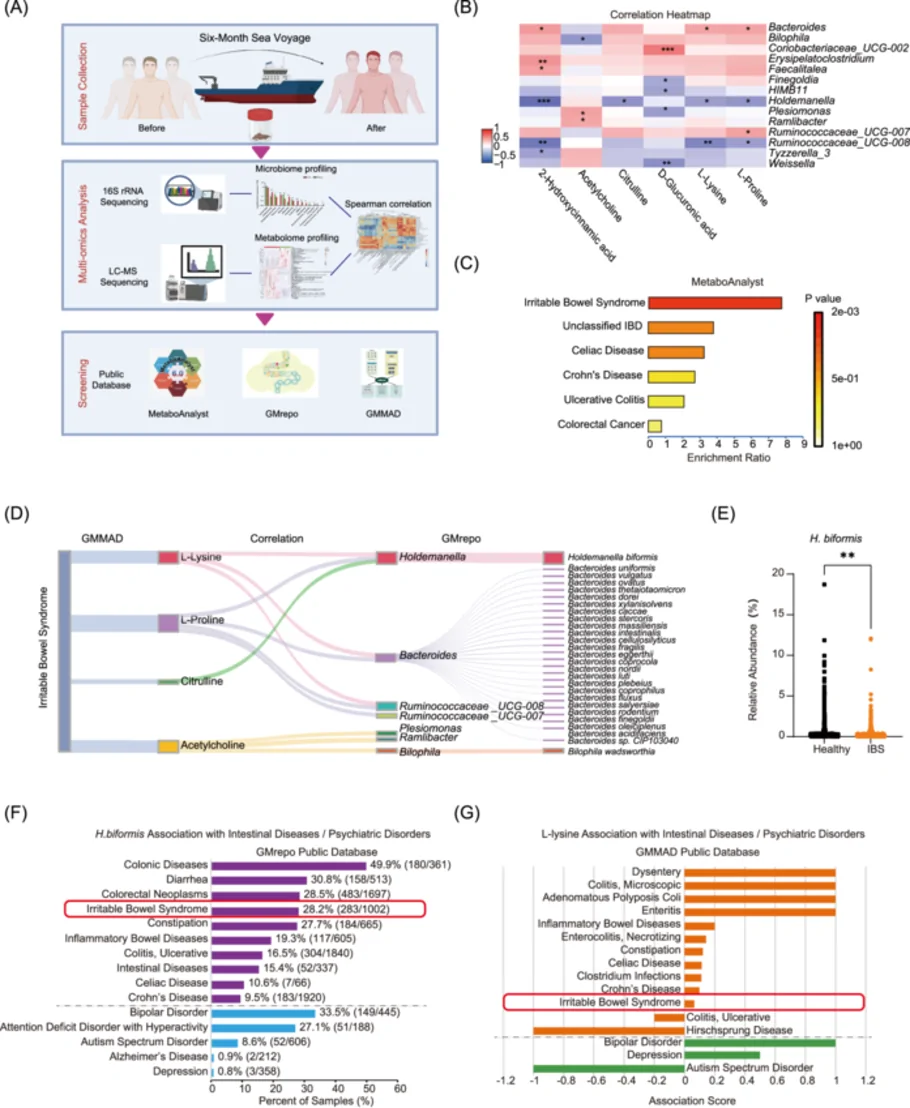
Integrated multi‐omics analysis of seafarers' gut microbiome and metabolome combined with public database mining. (A) Workflow of the analysis, including microbiome (16S rRNA sequencing) and metabolome (liquid chromatography‐mass spectrometry) profiling, multi‐omics integration, and disease association mining using public database. (B) Spearman correlations heatmap showing associations between six KEGG‐annotated key differential metabolites (columns) with their co‐varying microbial genera (rows). Colour intensity indicates correlation strength (red, positive; blue, negative). *p < 0.05; **p < 0.01; ***p < 0.001. (C) Pathway enrichment analysis (MetaboAnalyst 6.0) of intestinal diseases based on the six key differential metabolites, with IBS showing the highest enrichment ratio (enrichment ratio = 7.78, *p* = 0.02). (D) Sankey diagram illustrating the IBS‐associated networks: diseases (left), metabolites (middle), and microbes (right). l‐lysine and *H. biformis* were prioritised as candidate IBS‐related targets. Node sizes correspond to the cumulative association strength of each entity (diseases/metabolites/microbes). (E) Relative abundance of *H. biformis* between IBS patients (*n* = 470) and healthy controls (*n* = 1000), based on GMrepo database (*p* = 0.0013). (F) Associations between *H. biformis* abundance and various disease phenotypes, with purple representing intestinal diseases and blue representing psychiatric disorders (GMrepo database). (G) Association scores between l‐lysine and disease phenotypes, with orange indicating intestinal diseases and green indicating psychiatric disorders (GMMAD database). **p < 0.01. GMMAD, gut microbial metabolite association with disease; GMrepo, data repository for gut microbiota; *H. biformis*, *Holdemanella biformis*; IBS, irritable bowel syndrome; KEGG, Kyoto encyclopaedia of genes and genomes.

We previously conducted 16S rRNA gene sequencing and liquid chromatography‐mass spectrometry (LC‐MS) untargeted metabolomics on faecal samples from seafarers before and after long‐term voyages, identifying voyage‐associated shifts in both gut microbiota and metabolites [6]. To further delineate disease‐relevant alterations, we integrated these datasets with the human KEGG database to pinpoint key differential metabolites and their corresponding microbes. Given the high incidence of intestinal disorders during long‐term voyages [3, 4, 5], we systematically screened public resources, including MetaboAnalyst 6.0 (https://www.metaboanalyst.ca/), GMrepo (https://gmrepo.humangut.info/home), and GMMAD (http://guolab.whu.edu.cn/GMMAD), for links between the metabolic and microbial features and gut diseases (Figure 1A). Among the top 50 voyage‐associated metabolites, six (2‐Hydroxycinnamic acid, acetylcholine, citrulline, d‐glucuronic acid, l‐lysine, and l‐proline) mapped directly to human KEGG metabolic pathways. These were associated with 14 altered bacteria genera, including *Bacteroides*, *Bilophila*, and *Holdemanella* (Figure 1B and Table S1). Pathway enrichment analysis using MetaboAnalyst indicated that these metabolites were most strongly linked to IBS (enrichment ratio = 7.78, *p* = 0.02) (Figure 1C and Table S2), a condition frequently reported among seafarers [4, 5]. Next, GMMAD‐based screening further identified four of the six metabolites (l‐lysine, l‐proline, citrulline, and acetylcholine) as IBS‐related (Figure 1D and Table S3). Using GMrepo, we identified seven bacterial genera linked to these metabolites, of which *Holdemanella*, *Bacteroides*, and *Bilophila* contained species previously associated with IBS (Figure 1D and Table S4).

Given that l‐lysine is the only essential amino acid among the four metabolites and that *H. biformis* is the specific IBS‐associated species within its genus, we selected this pair for further investigation. Data pooled from GMrepo revealed a significantly lower abundance of *H. biformis* in individuals with IBS compared to healthy controls (*p* = 0.0013) (Figure 1E and Table S5). Moreover, both *H. biformis* and l‐lysine were associated with not only gastrointestinal diseases, including IBS, but also with neuropsychiatric disorders such as bipolar disorder, depression, and autism spectrum disorder (Figure 1F,G and Table S6–7). These findings prompted us to further explore the roles of l‐lysine and *H. biformis* in IBS pathogenesis, particularly in the context of gut‐brain axis dysfunction.

### l‐lysine improves intestinal barrier function and alleviates anxiety‐like behaviours in an IBS‐D mouse model

To model diarrhoea‐predominant irritable bowel syndrome (IBS‐D), mice were subjected to low‐dose acetic acid enemas combined with restraint stress, a protocol previously validated for stable IBS phenotypes (Figure S1A) [13, 14]. To optimise the model, we compared 1%, 2%, and 3% (v/w) acetic acid enemas. Both 2% and 3% acetic acid enemas combined with restraint stress significantly increased the faecal water content (*p* < 0.05) (Figure S1B), but 3% acetic acid enema led to a significant rise in mortality (*p* = 0.0033) (Figure S1C). Therefore, 2% acetic acid enemas combined with restraint stress was selected for all subsequent experiments.

Immunofluorescence staining of zonula occludens‐1 (ZO‐1), a tight junction protein encoded by *Tjp1*, revealed a significant reduction in ZO‐1 intensity in the ileum and colon of IBS‐D mice compared to controls (*p* < 0.05) (Figure S1D,E). Consistently, *Tjp1* mRNA levels were also lower in IBS‐D mice (*p* < 0.05) (Figure S1F). Intestinal permeability, assessed by serum FITC‐Dextran (MW4000), was significantly elevated in IBS‐D mice compared to the controls (*p* = 0.0050) (Figure S1G). The abdominal withdrawal reflex (AWR) showed increased visceral sensitivity in IBS‐D mice compared to controls, as reflected by higher AWR scores across inflation volumes (*p* = 0.0422) (two‐way ANOVA analysis) (Figure S1H). Together, these results validate the 2% acetic acid enemas combined with restraint stress protocol as a representative IBS‐D mouse model.

**FIGURE 2:**
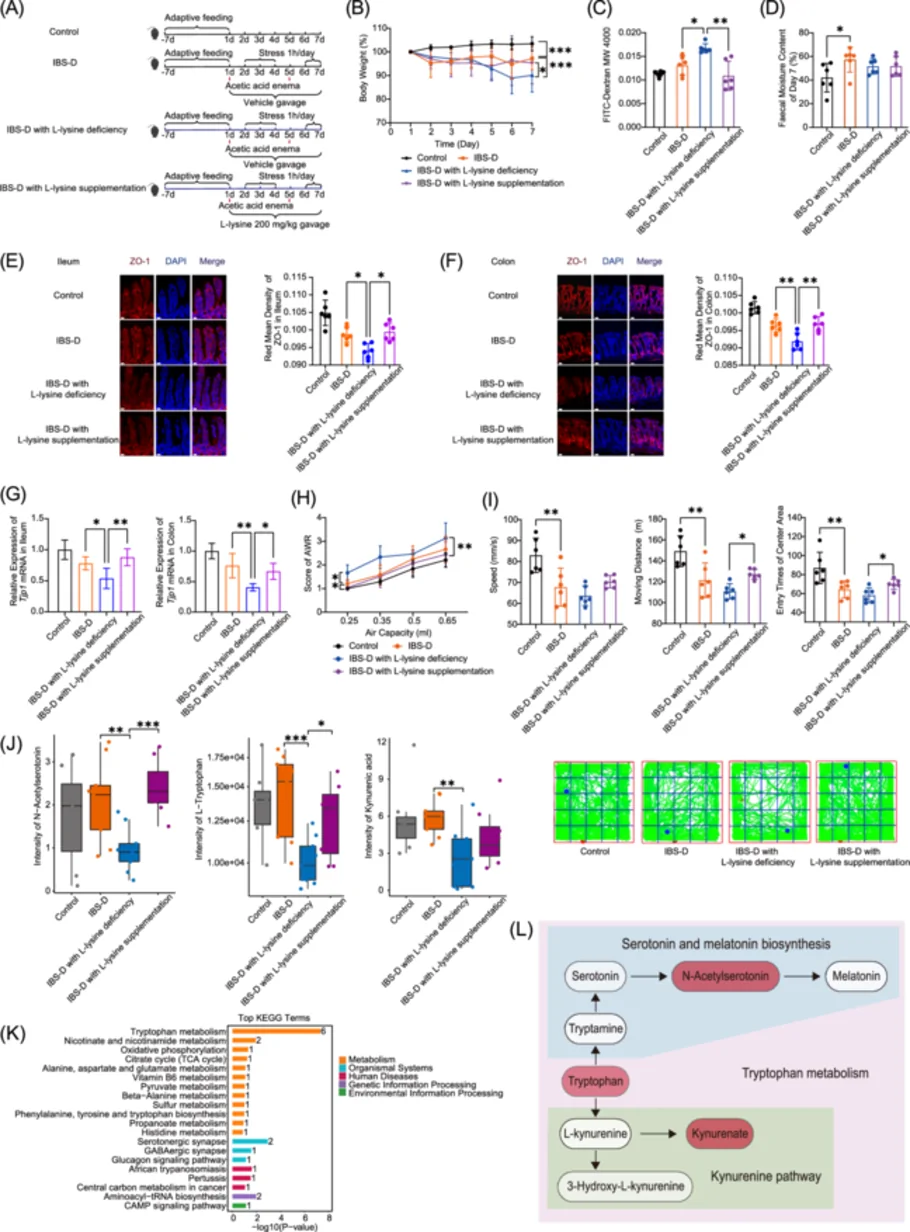
l‐lysine supplementation ameliorates intestinal barrier dysfunction and modulates anxiety‐like behaviours in IBS‐D model mice. (A) Mice experimental design. During a 7‐day adaptation period, mice in the control and IBS‐D groups were fed an AIN‐93M purified diet, while mice in the IBS‐D with l‐lysine deficiency and supplementation groups received l‐lysine‐deficient AIN‐93M. During the 7‐day intervention period, mice in the l‐lysine supplementation group were gavaged daily with l‐lysine (200 mg/kg). (B) Body weight changes. (C) Intestinal permeability was assessed by serum fluorescent value of FITC‐Dextran MW4000 levels. (D) Faecal moisture content. Representative immunofluorescence images of ZO‐1 (left) and red mean density analysis of ZO‐1 (right) in the ileum (E) and colon (F). Scale bar, 20 μm. (G) qPCR analysis of *Tjp1* mRNA expression in the ileum and colon (normalised to *Gapdh*). (H) Visceral hypersensitivity measured by AWR scores to colorectal distension. (I) Open field test behavioural analysis. Quantification of the speed (mm/s), moving distance (m), and entry times of centre area (frequency) during 30‐min test sessions. The green lines in the four grid diagrams below represent the movement trajectories of mice during the open field test. (J) Serum neurotransmitter levels. Intensity of N‐acetylserotonin, l‐tryptophan, and kynurenic acid in serum. (K) KEGG pathway enrichment analysis of serum differential neurotransmitters. (L) Schematic overview of changed neurotransmitter metabolic pathways. *p < 0.05; **p < 0.01; ***p < 0.001. AWR, abdominal withdrawal reflex; IBS‐D, diarrhoea‐predominant irritable bowel disorders; KEGG, Kyoto encyclopaedia of genes and genomes; qPCR, quantitative polymerase chain reaction; ZO‐1: zonula occludens‐1.

To investigate the role of l‐lysine, mice were assigned to four groups: control, IBS‐D, IBS‐D with l‐lysine deficiency, and IBS‐D with l‐lysine supplementation (Figure 2A). After 1‐week adaptation, l‐lysine‐deficient diets did not alter baseline body weight (Figure S1I), but following IBS‐D induction, mice on the deficient diet lost more weight than IBS‐D controls (*p* = 0.0006), which was partially reversed by l‐lysine supplementation (*p* = 0.0101) (mixed‐effects model analysis) (Figure 2B). Serum FITC‐Dextran levels were significantly elevated in l‐lysine‐deficient IBS‐D mice compared to IBS‐D alone (*p* = 0.0312), and was reduced upon l‐lysine supplementation (*p* = 0.0010) (Figure 2C), indicating improved barrier function.

Although faecal water content increased significantly over time in all IBS‐D groups (*p* < 0.05), it was not significantly altered by l‐lysine deficiency or supplementation (Figure 2D and Figure S1J). However, ZO‐1 density in the ileum and colon was further reduced by l‐lysine deficiency and significantly restored by supplementation (*p* < 0.05) (Figure 2E,F). Similarly, *Tjp1* expression was lowest in the l‐lysine deficient group and significantly elevated following supplementation (*p* < 0.05) (Figure 2G). These results reinforce the role of l‐lysine in preserving epithelial integrity.

l‐lysine also modulated visceral hypersensitivity. AWR scores were significantly higher in l‐lysine‐deficient IBS‐D mice compared to IBS‐D alone (*p* = 0.0452), and were reduced upon l‐lysine supplementation (*p* = 0.005) (Figure 2H). In open‐field tests, IBS‐D mice exhibited classic anxiety‐like behaviours, including reduced speed, movement, and central‐zone entries (*p* < 0.05), which tended to worsen with l‐lysine deficiency and were significantly alleviated by l‐lysine supplementation (*p* < 0.05, Figure 2I).

Targeted metabolomics of serum neurotransmitters revealed that levels of N‐acetylserotonin and l‐tryptophan were significantly lower in the l‐lysine‐deficient mice and restored with l‐lysine supplementation (*p* < 0.05) (Figure 2J). KEGG analysis identified tryptophan metabolism as the most significantly altered pathway (Figure 2K), particularly two sub‐pathways: serotonin/melatonin biosynthesis and the kynurenine pathway (Figure 2L). These findings suggest that l‐lysine alleviates anxiety‐like behaviour in IBS‐D mice, potentially via modulation of tryptophan metabolism and gut‐brain axis function.

### *H. biformis* **alleviates intestinal barrier dysfunction but aggravates anxiety‐like behaviours**

**FIGURE 3:**
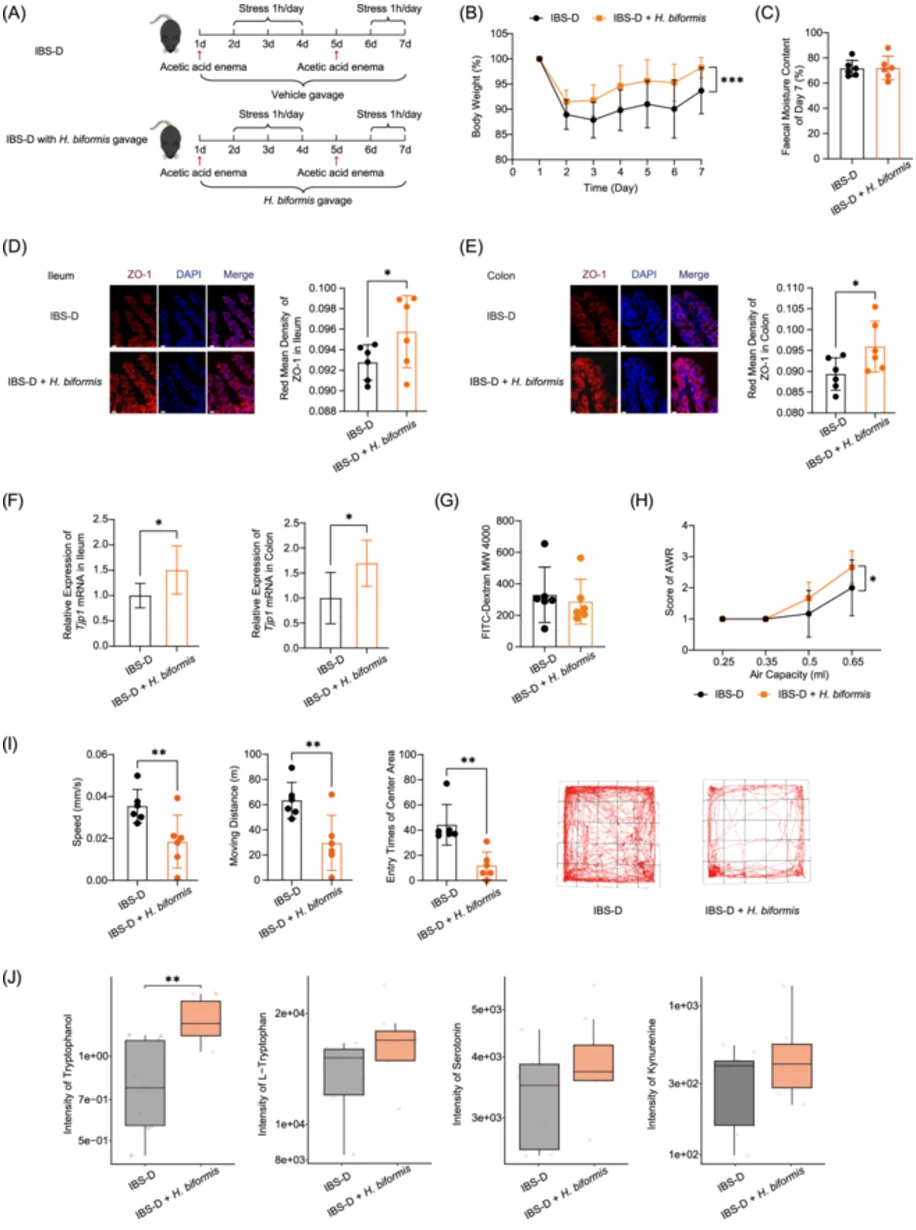
Effects of *H. biformis* on the intestinal barrier function and behaviours in IBS‐D model mice. (A) Mice experimental design. Germ‐free mice in the IBS‐D with *H. biformis* gavage group were gavaged with *H. biformis* suspension (10^8^ CFU/mL, 0.2 mL) per day for seven consecutive days, while germ‐free mice in the IBS‐D group were gavaged with vehicle daily. (B) Body weight changes. (C) Faecal moisture content on Day 7. Representative immunofluorescence images of ZO‐1 (left) and red mean density analysis of ZO‐1 (right) in the ileum (D) and colon (E). Scale bar, 20 μm. (F) qPCR analysis of *Tjp1* mRNA expression in the ileum and colon (normalised to *Gapdh*). (G) Intestinal permeability was assessed by serum fluorescent value of FITC‐Dextran MW4000 levels. (H) Visceral hypersensitivity measured by AWR scores to colorectal distension. (I) Open field test behavioural analysis. Quantification of the speed (mm/s), moving distance (m), and entry times of centre area (frequency) during 30‐min test sessions. The red lines in the two grid diagrams on the right show the movement trajectories of germ‐free mice during the open field test. (J) Serum neurotransmitter levels. Intensity of tryptophanol, l‐tryptophan, serotonin, and kynurenine in serum. *p < 0.05; **p < 0.01.

To assess whether *H. biformis* can colonise the gut of germ‐free mice, we performed microscopic analysis of intestinal content smears, following procedures from previous studies [15, 16]. Smears obtained before and intragastric administration of *H. biformis* (10^8^ CFU/mL × 0.2 mL, given twice over 1 week) showed no detectable *H. biformis* (Figure S2A–D), consistent with earlier reports that *H. biformis* cannot stably colonise germ‐free mice [17]. To investigate its functional effects, we administrated *H. biformis* (0.2 mL × 10^8^ CFU/mL) daily for 1 week to germ‐free C57BL6/J mice subjected to IBS‐D induction (Figure 3A). Gavage with *H. biformis* significantly ameliorated weight loss in IBS‐D germ‐free mice (*p* = 0.0002, mixed‐effects model; Figure 3B) but did not affect faecal water content (Figure 3C). Immunofluorescence staining revealed significantly increased ZO‐1 expression in the ileum and colon (*p* < 0.05) (Figure 3D,E), and *Tjp1* mRNA levels were similarly upregulated (*p* < 0.05) (Figure 3F), indicating improved epithelial tight junction integrity. However, serum FITC‐Dextran levels were not significantly altered (Figure 3G), suggesting only partial restoration of barrier function.

Despite these intestinal improvements, *H. biformis* worsened visceral sensitivity. AWR scores were significantly higher in *H. biformis*‐treated mice relative to untreated IBS‐D germ‐free controls (*p* = 0.0446) (Figure 3H). Moreover, open‐field tests showed pronounced anxiety‐like behaviours following *H. biformis* administration, including reduced locomotor speed, shorten travel distances, and fewer entries into the centre zone (*p* < 0.05) (Figure 3I). Targeted metabolomics showed a significant increase in serum tryptophanol (*p* < 0.01) (Figure 3J). KEGG pathway analysis revealed that tryptophan metabolism was the primary altered pathway (*p* = 0.02578) (Table S8), linking the observed behavioural changes to microbial modulation of host neurochemistry.

Together, these findings suggest that *H. biformis* partially restores intestinal barrier function in IBS‐D mice but may simultaneously exacerbate anxiety‐like behaviours through tryptophan pathway perturbations.

### **l‐lysine and** *H. biformis* **enhance ZO‐1 cell tight junctions and promote epithelial wound healing**

**FIGURE 4:**
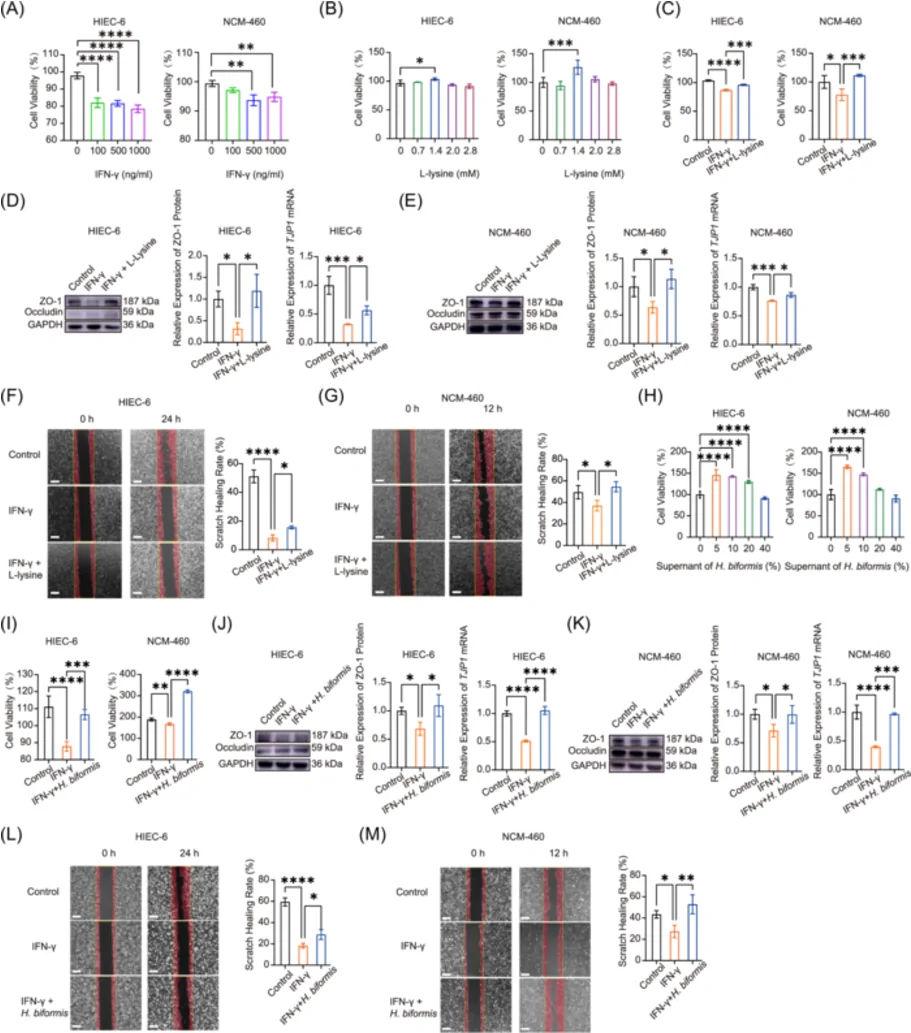
l‐lysine and *H. biformis* supernatant enhance tight junction integrity and promote wound healing in intestinal epithelial cells. (A) dose–response effects of IFN‐γ (0, 100, 500, and 1000 ng/mL) on the viability of HIEC‐6 and NCM‐460 cells after 24 h treatment, measured by CCK‐8 assay. (B) dose–response effects of l‐lysine (0, 0.7, 1.4, 2.0, and 2.8 mM) on the viability of HIEC‐6 and NCM‐460 cells. (C) Protective effect of 1.4 mM l‐lysine against IFN‐γ pre‐processed HIEC‐6 and NCM‐460 cell viability. Effects of 1.4 mM l‐lysine intervention on the ZO‐1 and Occludin expression of IFN‐γ pre‐processed HIEC‐6 (D) and NCM‐460 (E) cells by western blot and quantitative polymerase chain reaction (qPCR) (normalised to GAPDH). Effects of 1.4 mM l‐lysine intervention on the scratch injury healing rate of IFN‐γ pre‐processed HIEC‐6 (F) and NCM‐460 (G) cells. Left: Phase‐contrast images at 0, 12, and 24 h post‐scratching (Scale bar, 100 μm); Right: Quantified healing rates. (H) Dose–response effects of *H. biformis* supernatant (0, 5%, 10%, 20%, and 40%) on the viability of HIEC‐6 and NCM‐460 cells (24 h; CCK‐8). (I) Protective effect of 5% *H. biformis* supernatant against IFN‐γ pre‐processed HIEC‐6 and NCM‐460 cell viability. Effects of 5% *H. biformis* supernatant on the ZO‐1 and Occludin expression of IFN‐γ pre‐processed HIEC‐6 (J) and NCM‐460 (K) cells by western blot and qPCR (normalised to GAPDH). Effects of 5% *H. biformis* supernatant on the scratch injury healing rate of IFN‐γ pre‐processed HIEC‐6 (L) and NCM‐460 (M) cells. Left: Phase‐contrast images at 0, 12, and 24 h post‐scratching (Scale bar, 100 μm); Right: Quantified healing rates. *p < 0.05; **p < 0.01; ***p < 0.001; ****p < 0.0001.

Previous studies have shown that IFN‐γ is significantly elevated in the serum and tissues of IBS patients and can induce intestinal epithelial barrier dysfunction [18, 19]. Therefore, in this study, IFN‐γ pretreatment of HIEC‐6 and NCM‐460 cells was performed to simulate intestinal epithelial barrier injury. Different concentrations of IFN‐γ were added to screen for the optimal dose for reducing HIEC‐6 and NCM‐460 cell viability. The results showed that 100 ng/mL and 500 ng/mL IFN‐γ significantly inhibited HIEC‐6 and NCM‐460 cells, respectively (*p* < 0.01) (Figure 4A).

Next, various concentrations of l‐lysine were added to the culture media of HIEC‐6 cells and NCM‐460 cells for 24 h to determine the appropriate intervention dose. l‐lysine had no inhibitory effect on cell viability and promoted cell activity at a concentration of 1.4 mM (*p* < 0.05) (Figure 4B). l‐lysine also significantly reversed the inhibitory effect of IFN‐γ on HIEC‐6 and NCM‐460 cell viability (*p* < 0.001) (Figure 4C). IFN‐γ significantly reduced ZO‐1 expression in both cell lines (*p* < 0.01), while l‐lysine markedly restored ZO‐1 levels (*p* < 0.05) (Figure 4D,E). A scratch assay was performed to assess the effect of l‐lysine on the wound healing ability of IFN‐γ pretreated cells. IFN‐γ significantly impaired healing in HIEC‐6 and NCM460 cells (*p* < 0.05), and this effect was significantly alleviated by l‐lysine (*p* < 0.05) (Figure 4F,G). These findings indicate that l‐lysine improves ZO‐1‐mediated tight junctions and promotes wound healing in intestinal epithelial cells.

As *H. biformis* is an obligate anaerobic bacterium and cannot be cocultured with cells, we used *H. biformis* supernatant as an alternative. Different concentrations of *H. biformis* supernatant were added to epithelial cell cultures. A 5% *H. biformis* supernatant concentration significantly increased the viability of HIEC‐6 and NCM460 cells (*p* < 0.0001) (Figure 4H). The supernatant also significantly alleviated the inhibitory effect of IFN‐γ on the viability of both cell lines (*p* < 0.001) (Figure 4I) and greatly enhanced ZO‐1 expression (*p* < 0.05) (Figure 4J,K). Scratch assay further demonstrated that *H. biformis* supernatant significantly rescued the IFN‐γ‐induced suppression of epithelial cell healing (*p* < 0.05) (Figure 4L,M). Together, these findings indicate that *H. biformis* improves ZO‐1 tight junctions and enhances wound healing of human intestinal epithelial cells.

### *H. biformis* **can metabolise l‐lysine while l‐lysine inhibits the growth of** *H. biformis*

**FIGURE 5:**
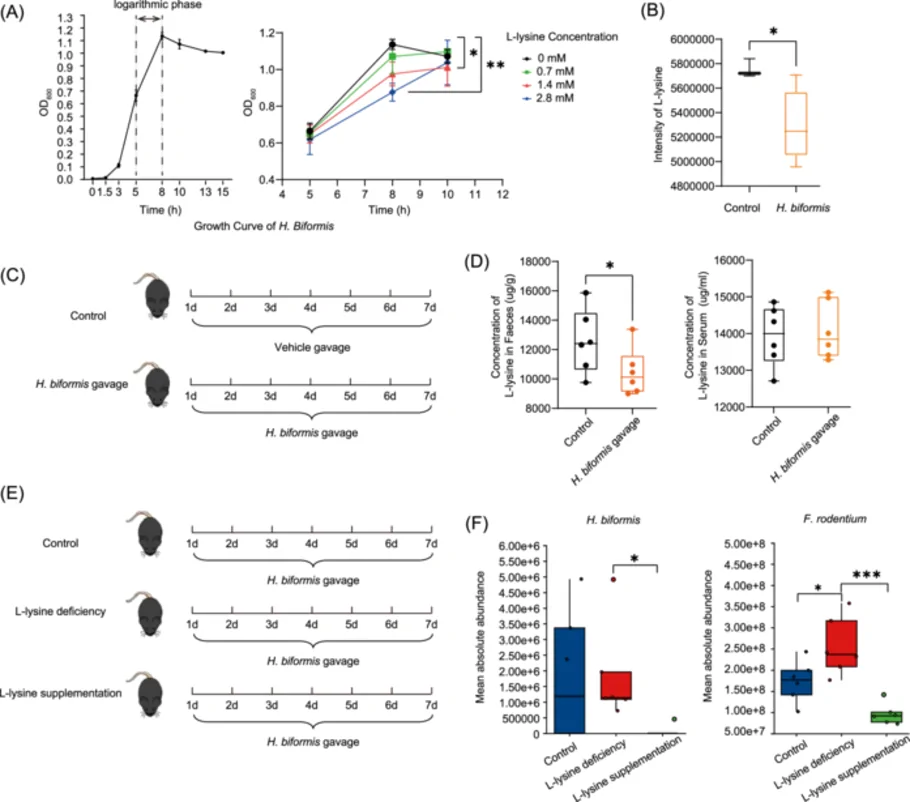
Interaction between *H. biformis* and l‐lysine *in vitro* and in vivo experiments. (A) l‐lysine dose‐dependently inhibits *H. biformis* growth in vitro. Growth curves of *H. biformis* measured by OD_600_ (left); dose–response effects of l‐lysine (0, 0.7, 1.4, and 2.8 mM) on the growth curve of *H. biformis* (right). (B) Intensity of l‐lysine between the control medium and the medium containing *H. biformis*. (C) Mice experimental design. For seven consecutive days, mice in the *H. biformis*‐gavage group were gavaged with *H. biformis* suspension (10^8^ CFU/mL, 0.2 mL) daily. (D) Intensity of l‐lysine in the faeces and serum between *H. biformis*‐gavage group and control group. (E) Mice experimental design. All groups (control, l‐lysine deficiency, and l‐lysine supplementation groups) were gavaged with *H. biformis* suspension (10^8^ CFU/mL, 0.2 mL) per day for seven consecutive days. (F) Mean absolute abundance of *H. biformis* (left) and *F. rodentium* (right) among control, l‐lysine deficiency, and l‐lysine supplementation groups. *F. rodentium*: *Faecalibaculum rodentium*.

Growth curve analysis revealed that *H. biformis* entered a logarithmic growth phase between 3 and 8 h. l‐lysine supplementation at concentrations of 1.4 mM and 2.8 mM significantly inhibited bacterial growth at 8 h (*p* < 0.05) (Figure 5A), consistent with previous multi‐omics results showing a negative correlation between l‐lysine and *H. biformis* abundance in vitro (Figure 1B). Moreover, the concentration of l‐lysine in *H. biformis*‐containing medium was significantly reduced compared to control cultures, indicating that *H. biformis* actively metabolises l‐lysine (Figure 5B).

To assess this relationship in vivo, we measured l‐lysine concentrations in the faeces and serum of the mice gavaged with *H. biformi*s or vehicle control (Figure 5C). Faecal l‐lysine levels were significantly lower in the *H. biformis*‐treated group than in controls (*p* = 0.043) (Figure 5D), further supporting microbial consumption of l‐lysine. Conversely, the abundance of *H. biformis* in the faeces was qualified in three mice groups (l‐lysine‐deficient, l‐lysine‐supplemented, and controls), each of which received *H. biformis* via intragastric gavage (Figure 5E). Absolute quantification of 16S rRNA gene sequencing revealed a significant reduction in *H. biformis* abundance in the l‐lysine supplemented group compared to the l‐lysine‐deficient group (*p* < 0.05) (Figure 5F).

Given that *H. biformis* cannot stably colonise the murine gut, we investigate its murine homologue, *Faecalibaculum rodentium* (*F. rodentium*), to further examine the in vivo relationship [17]. As expected, l‐lysine deficiency significantly increased the abundance of *F. rodentium* in mice compared to controls, whereas l‐lysine supplementation markedly reduced its abundance of *F. rodentium* compared to the deficient group (*p* < 0.05) (Figure 5F).

Together, these results demonstrate a reciprocal relationship between l‐lysine and *H. biformis*: while *H. biformis* actively metabolises l‐lysine, elevated l‐lysine levels suppress its growth.

### *H. biformis* metabolises l‐lysine via the lysine degradation pathway, while l‐lysine inhibits *H. biformis* growth by downregulating carbohydrate and energy metabolism

**FIGURE 6:**
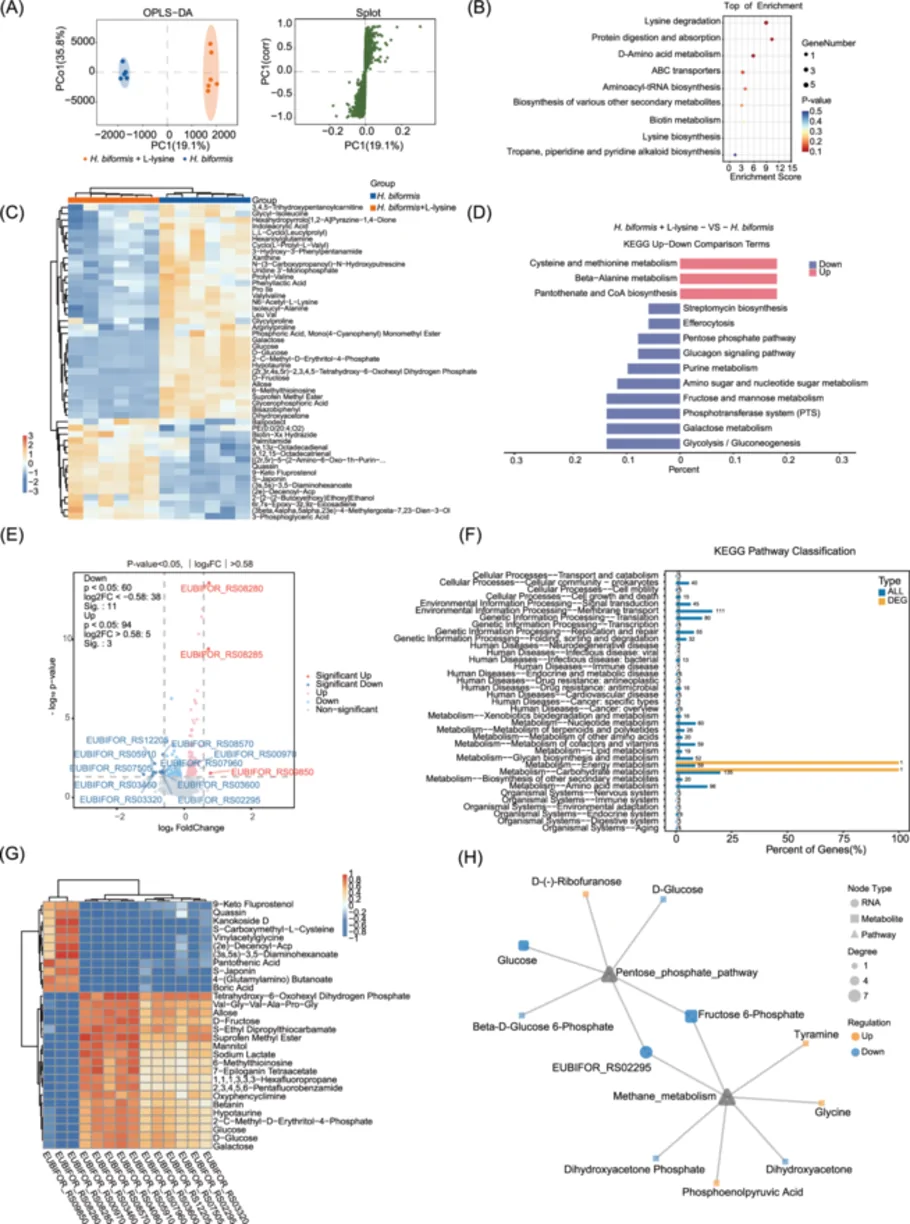
Untargeted metabolomics and prokaryotic RNA‐seq analysis of *H. biformis* pellets from cultures with or without l‐lysine. (A) Multivariate analysis of metabolic profiles. OPLS‐DA (left) and OPLS‐DA‐Splot (right) analysis showing metabolic differences between groups. (B) Bacterial Kyoto encyclopaedia of genes and genomes (KEGG) enrichment analysis of lysine‐related metabolic pathways. (C) Heatmap of the top 50 differential metabolites between groups. (D) KEGG pathways enrichment analysis of the identified differential metabolites. (E) Prokaryotic RNA‐seq analysis of differentially expressed genes between groups. Volcano plot illustrating significantly upregulated and downregulated genes. (F) KEGG pathway enrichment analysis of differentially expressed genes. (G) Spearman correlation analysis between differential metabolites (row) and expressed genes (column). (H) KGML analysis of differential metabolites and expressed genes. Integrated visualisation of metabolite‐gene interactions. OPLS‐DA, orthogonal partial least squares‐discriminant analysis; KGML, Kyoto encyclopaedia of genes and genomes markup language.

To explore the underlying metabolic interactions between *H. biformis* and l‐lysine, we conducted untargeted metabolomics using LC/gas chromatography‐MS (LC/GC‐MS) and performed prokaryote RNA‐seq on bacterial pellets from cultures with or without l‐lysine. Principal component analysis (PCA), orthogonal partial least‐squares‐discriminant analysis (OPLS‐DA), and OPLS‐DA‐Splot confirmed clear metabolic distinctions between the two groups (Figure 6A and Figure S3A). KEGG pathway enrichment analysis revealed that the lysine degradation pathway exhibited the highest enrichment score in *H. biformis* (enrichment score = 8.91, *p* = 0.0002) (Figure 6B), indicating this as the principal route for l‐lysine metabolism.

Among the top 50 differential metabolites, 68% (34/50) were significantly downregulated in the presence of l‐lysine (Figure 6C). KEGG pathway analysis further showed that l‐lysine exposure led to significant downregulation of core energy‐yielding processes in *H. biformis*, including glycolysis, the pentose phosphate pathway, and the phosphotransferase system (Figure 6D). Prokaryote RNA‐seq identified significant changes in the expression of key genes (e.g., *EUBIFOR_RS02295, EUBIFOR_RS12205, EUBIFOR_RS08285*) following l‐lysine coculture (Figure 6E), and KEGG gene pathway analysis confirmed that pathways related to carbohydrate and energy metabolism were broadly suppressed (Figure 6F). These findings suggest that l‐lysine may inhibit *H. biformis* growth by disrupting its central metabolic pathways in vitro.

To further elucidate metabolite‐gene relationships, Spearman correlation analysis was performed between differential metabolites and differentially expressed genes in *H. biformis*. The top 30 metabolites were significantly correlated with 14 differentially expressed genes (Figure 6G). KGML network analysis identified glucose, fructose 6‐phosphate, tyramine, and other key metabolites has been altered by l‐lysine, which also downregulated the expression of *EUBIFOR_RS02295* and suppressed the pentose phosphate (*p* < 0.05) and methane metabolism pathways (*p* = 0.0321) that are central to carbohydrate and energy metabolism in *H. biformis* (Figure 6H). Additionally, untargeted metabolomics of *H. biformis* culture supernatant demonstrated a significant increase in butyric acid levels (*p* < 0.01) (Figure S3B), a short‐chain fatty acid known to support intestinal barrier integrity [20, 21].

Collectively, these data indicate that *H. biformis* metabolises l‐lysine primarily via lysine degradation pathway, while l‐lysine inhibits the growth of *H. biformis*, likely through suppression of carbohydrate and energy metabolism.

## 3 DISCUSSION

**FIGURE 7:**
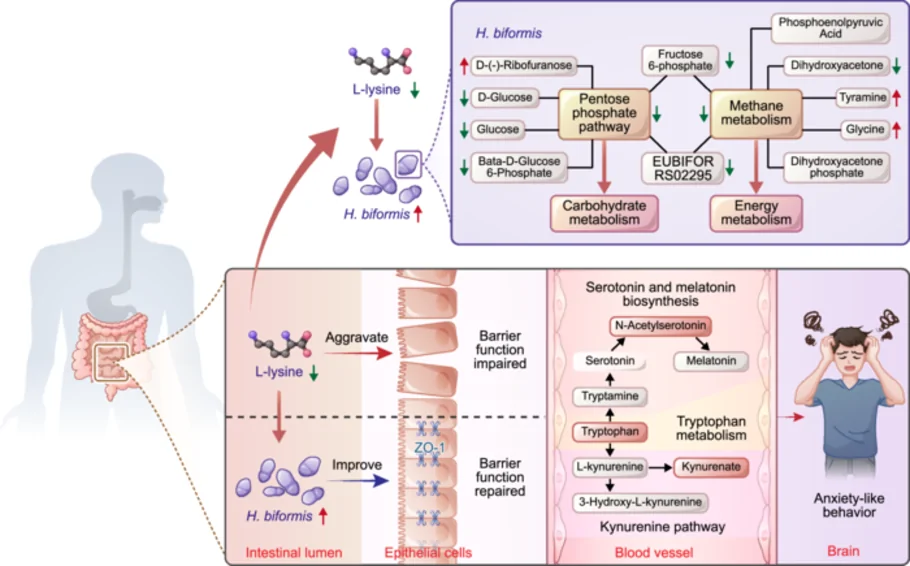
Schematic overview of the interaction between l‐lysine and *H. biformis* in regulating intestinal barrier function and anxiety‐like behaviour. A decrease in l‐lysine promotes *H. biformis* growth, which is beneficial for the reconstruction of impaired intestinal barrier function. Both l‐lysine deficiency and *H. biformis* growth aggravate anxiety‐like behaviours. l‐lysine inhibits the growth of *H. biformis*, potentially by the downregulation of its carbohydrate and energy metabolism pathways.

Previous studies have reported associations between disturbances in l‐lysine levels or *H. biformis* abundance and the pathogenesis of IBS [7, 8, 9, 11, 12]. Our multi‐omics investigation reveals a complex interaction between l‐lysine and *H. biformis* in the context of IBS, offering novel insights into microbe‐metabolite dynamics and their implications for therapeutic intervention. This study presents three key findings: (1) l‐lysine exerts protective effects on both intestinal barrier function and anxiety‐like symptoms in IBS‐D; (2) *H. biformis* exhibits paradoxical effects, improving intestinal barrier integrity while exacerbating anxiety‐like behaviours; and (3) a bidirectional metabolic relationship exists between the two, with l‐lysine inhibiting the growth of *H. biformis*, which in turn metabolises l‐lysine (Figure 7).

The role of l‐lysine in maintaining intestinal homoeostasis is particularly noteworthy. Our results demonstrate that l‐lysine supplementation significantly upregulates ZO‐1 expression in both the ileum and colon, reduces intestinal permeability, and alleviates visceral hypersensitivity in IBS‐D mice. These findings align with the evidence highlighting the importance of l‐lysine in the restoration of intestinal epithelial barrier function and tissue repair [7]. Furthermore, the observed alleviation in anxiety‐like behaviours and normalisation of tryptophan metabolism following l‐lysine supplementation suggest its regulatory role on the gut‐brain axis, likely through modulation of serotonin and kynurenine pathways. Tryptophan metabolism plays a central role in IBS pathogenesis [22]: reduced serotonin levels impairs intestinal motility, contributing to IBS symptoms such as diarrhoea and constipation [23], while dysregulated kynurenine pathway activation may trigger intestinal inflammation, visceral hypersensitivity, and physiological comorbidities [24, 25]. The ability of l‐lysine to normalise ureagenesis in IBS rats [26], may further support this systemic detoxification role. These findings position l‐lysine as a promising, dual‐action therapeutic candidate for IBS‐related gastrointestinal and psychological symptoms.

In contrast, the effects of *H. biformis* more nuanced. While *H. biformis* enhanced tight junction protein expression and promoted wound healing in vitro, consistent with its known butyrogenic properties [17], its administration to germ‐free IBS‐D mice exacerbated anxiety‐like behaviours and visceral hypersensitivity. This dichotomy reflects the complexity of gut‐brain crosstalk, wherein microbial metabolites that benefit intestinal epithelial integrity may simultaneously exert unintended effects on host neurophysiology. The role of *Holdemanella* on psychological disorders remains controversial. Some studies suggest a protective effect against depression and anxiety [27, 28, 29], while others have linked *Holdemanella* to neurodevelopment and behavioural disorders such as autism spectrum disorder [30], obsessive‐compulsive behaviour [31], and diabetic cognitive impairment [32]. Notably, most prior studies were correlational, based on omics data alone. In contrast, our use of germ‐free IBS‐D model provides causal evidence linking *H. biformis* to worsened anxiety‐like behaviours. Our neurotransmitter metabolomics data implicate tryptophan metabolic dysregulation as a potential mechanism. Given these findings, the therapeutic application of *H. biformis*, despite its intestinal benefits, should be approached with caution in clinical settings, particularly in patients with stress‐sensitive or neuropsychiatric comorbidities [33, 34].

The discovery of a reciprocal metabolic relationship between l‐lysine and *H. biformis* adds another layer of complexity to our understanding of intestinal microbial ecology. Our multi‐omics analyses revealed that *H. biformis* actively metabolises l‐lysine primarily through lysine degradation pathways, while l‐lysine inhibits *H. biformis* growth by downregulating its carbohydrate and energy metabolism. This antagonistic interaction likely underlies the negative correlation observed in our initial omics data and suggests the existence of a feedback regulatory mechanism within the intestinal ecosystem. Moreover, the in vivo evidence that l‐lysine supplementation reduces abundance of *F. rodentium*, a murine homolog of *H. biformis*, further supports this reciprocal relationship.

Although gut microbiota and their metabolites have been increasingly implicated in host health and diseases [35, 36, 37], studies investigating direct interactions between specific microbes and metabolites remain limited. Our findings demonstrate that under stressful conditions, both l‐lysine and *H. biformis* contribute to the restoration of impaired intestinal barrier function. A decrease in l‐lysine promotes *H. biformis* growth, aiding epithelial repair, despite potential adverse neurological effects. Overall, this study advances our understanding of microbe–metabolite interaction in IBS and emphasises their importance in guiding probiotic development and nutritional intervention.

From a translational perspective, our findings suggest multiple therapeutic opportunities. l‐lysine supplementation may offer a dual benefit in alleviating both intestinal and psychological symptoms in IBS. The butyrogenic activity of *H. biformis* may also be harnessed for intestinal barrier repair, although its neurological effects must be carefully considered. Additionally, targeting the lysine degradation pathway in *H. biformis* could provide a novel approach to modulate this abundance or activity in a host‐specific manner.

Nonetheless, several limitations warrant attention. The exact molecular mechanisms by which *H. biformis* affects the intestine and neurological function remain unclear. Additionally, this study primarily focused on the interaction between l‐lysine and *H. biformis*, and the specific role of *EUBIFOR_RS02295* in mediating the inhibitory effect of l‐lysine on *H. biformis* growth requires further investigation. Future studies should aim to clarify these mechanisms and explore broader microbial‐metabolite networks relevant to IBS pathophysiology.

## 4 CONCLUSION

This study elucidates a complex metabolic interplay between l‐lysine and *H. biformis*, providing novel insights into their opposing yet complementary roles in the pathophysiology of IBS. l‐lysine deficiency aggravates intestinal barrier dysfunction but may indirectly promote repair by facilitating the growth of *H. biformis*, a symbiotic bacterium capable of enhancing epithelial integrity. However, both l‐lysine deficiency and *H. biformis* expansion are associated with increased anxiety‐like behaviours in IBS‐D. Mechanistically, l‐lysine suppresses the growth of *H. biformis*, while *H. biformis* metabolises l‐lysine, suggesting a bidirectional regulatory loop within the gut microenvironment. Under stress conditions, decreased l‐lysine availability may favour *H. biformis* proliferation, aiding intestinal barrier restoration at the possible expense of worsened psychological symptoms. These findings underscore the importance of precisely modulating host‐microbe metabolic interactions in IBS management. While l‐lysine supplementation may offer dual benefits for both gut integrity and mental health, the potential anxiety‐inducing effects of *H. biformis* warrants caution in its use as a probiotic.

## 5 MATERIAL AND METHODS

The overall design of this study was as follows (Figure S4): First, l‐lysine and *H. biformis* were identified as IBS‐related factors based on our previous multi‐omics data from human samples, along with analyses from public databases including KEGG, MetaboAnalyst 6.0, GMrepo, and GMMAD. Second, the individual effects of l‐lysine and *H. biformis* were examined in IBS mouse models, as well as their impact on the barrier function of intestinal epithelial cells. Finally, the interaction was investigated in in vitro and in vivo experiments to explore the potential mechanism.

### Animal experiment

#### Specific pathogen‐free (SPF) C57BL/6J mice and treatment

SPF C57BL/6J mice (equal numbers of males and females) were purchased from Beijing Sibeifu Biotechnology Co., Ltd. All mice were housed in the SPF‐grade Animal Experiment Centre of Changhai Hospital. Environmental conditions were maintained at a temperature of 22 ± 1°C, relative humidity of 55%–65%, and a 12‐h light/dark light cycle. Mice had ad libitum access to water and standard chow.

#### Investigate the interaction between l‐lysine and *H. biformis*

##### Part I In vivo effects of *H. biformis* on l‐lysine

A total of 12 mice were randomly divided into two groups (*n* = 6 per group). For seven consecutive days, mice in the control group received daily oral gavage with vehicle (phosphate‐buffered saline with 20% glycerol), while mice in the *H. biformis* group received daily gavage with *H. biformis* suspension (10^8^ CFU/mL, 0.2 mL).

##### Part II In vivo effects of l‐lysine on *H. biformis*

A total of 18 mice were randomly divided into three groups: control, l‐lysine deficiency and l‐lysine supplementation (*n* = 6 per group). All mice were gavaged with *H. biformis* suspension (10^8^ CFU/mL, 0.2 mL) once daily for seven consecutive days.

*H. biformis* was anaerobically cultured in chopped meat carbohydrate (CMC) medium. Bacteria in the logarithmic phase were harvested by centrifugation and resuspended in PBS to a final concentration of 10^8^ CFU/mL.

#### Impact of l‐lysine on intestinal barrier function and behaviours in IBS‐D mice

A total of 24 mice were randomly divided into four groups (*n* = 6 per group): control group, IBS‐D group, IBS‐D with l‐lysine deficiency, and IBS‐D with l‐lysine supplementation. During the 7‐day adaptation period, mice in the control and IBS‐D groups were fed an AIN‐93M purified diet, while those in the l‐lysine deficiency and supplementation groups received an l‐lysine‐deficient diet based on AIN‐93M. During the subsequent 7‐day intervention period, mice in the l‐lysine supplementation group were orally gavaged with l‐lysine (200 mg/kg/day), while mice in the IBS‐D group and l‐lysine deficiency groups received daily gavage with vehicle. IBS‐D was induced by administration of 2% v/w acetic acid enemas on Days 1 and 5, combined with 1‐h restraint stress on Days 2–4 and 6–7.

#### Germ‐free C57BL/6J mice and treatment

Germ‐free C57BL/6J mice (equal numbers of males and females) were purchased from GemPharmatech Co. Ltd and maintained in contamination‐controlled flexible film isolators. The germ‐free status of the mice was confirmed by aerobic and anaerobic culture, as well as Gram staining of faecal samples. A total of 12 germ‐free mice were randomly divided into two groups (*n* = 6 per group): IBS‐D group and IBS‐D with *H. biformis* gavage group. Mice in the *H. biformis*‐treated group were gavaged daily with *H. biformis* suspension (10^8^ CFU/mL, 0.2 mL) for seven consecutive days. All the germ‐free mice were subjected to 1% (v/w) acetic acid enemas on Days 1 and 5, combined with 1‐h restraint stress on Days 2–4 and 6–7 to induce IBS‐D.

At the endpoint, blood samples were collected and centrifuged (3000 rpm, 5 min, 4°C) to obtain serum for neurotransmitter analysis. Fresh faecal samples were collected using sterile tubes and stored at −80°C for 16S rRNA gene sequencing and metabolomics analysis.

#### Faecal moisture content detection

Fresh faecal samples were collected to determine both wet and dry weights. The faecal moisture content was calculated using the following formula: Faecal moisture content (%) = (Wet weight − Dry weight) × 100%/Wet weight.

#### Intestinal mucosal permeability detection

Intestinal permeability was determined by measuring the concentration of FITC‐Dextran MW4000 (HY‐128868A, MedChemExpress) in mouse serum. An aqueous solution of FITC‐Dextran was prepared at a dosage of 0.6 mg/g body weight in a total volume of 0.2 mL. Mice were fasted for 3 h before the end of the experiment, then weighed and gavaged with the prepared FITC‐Dextran solution. Blood samples were collected 3 h after gavage and centrifuged to isolate serum. A standard curve for FITC‐Dextran was generated, and the serum concentrations were calculated accordingly. Results were normalised by dividing the serum concentration by the mouse's body weight, and this normalised value was used for intergroup comparisons.

#### Immunohistochemistry and mean density analysis

The ileums and colon tissues were collected from mice, fixed in 4% phosphate‐buffered paraformaldehyde, and embedded in paraffin. Immunohistochemical staining was performed to assess the localisation and expression level of the tight junction protein ZO‐1. Tissue sections were processed using standard protocols. Mean density analysis of ZO‐1 was conducted using Aipathwell software (developed by Wuhan Servicebio Technology Co.) [38], which was used to scan and quantify the staining density in the tissue sections.

#### AWR experiment

Before the AWR experiment, mice were fasted for 24 h with free access to water. A urinary catheter with an airbag was lubricated with paraffin oil and gently inserted approximately 2 cm into the colon of each anesthetised mouse. The airbag was secured at the base of the tail. After recovery from anaesthesia, mice were allowed to acclimate to the inserted airbag for 30 min. Graded volumes of air (0.25, 0.35, 0.5, and 0.65 mL, respectively) were then rapidly inflated into the airbag for 20 s, with 30‐second intervals between inflations. Each target volume was tested three times per mouse, and behavioural responses were scored according to the AWR scale. The average of the three scores for each volume was recorded. A 5‐min interval was maintained between each volume administration. The AWR scoring criteria were as follows [39]: 0, no response to expansion; 1, slight head movement; 2, contraction of abdominal muscles; 3, abdominal wall lifting; 4, arching of the body and elevation of the pelvis.

#### Open field test [40]

The open field test was conducted over four consecutive days. The first 3 days served as an adaptation period, during which each mouse was gently placed on the experimenter's hand and allowed to move freely while the head and neck were lightly stroked. The duration of interaction was 5 min per mouse per day. On the fourth day, formal testing was performed. Each mouse was gently removed from its home cage and immediately placed in the centre of the open field apparatus. Animal behaviour was recorded for 30 min using automated behavioural analysis software. The following behavioural parameters were assessed: locomotor speed, distance travelled within the central area, and number of entries into the centre area.

#### Targeted metabolomic analysis of neurotransmitters

Targeted analysis of neurotransmitters in mouse serum was performed using ultra‐performance LC‐electrospray ionisation‐tandem MS (UPLC‐ESI‐MS/MS). Neurotransmitter metabolites were detected and quantified using multiple reaction monitoring (MRM) mode. For each analyte, the MRM transitions, declustering potentials (DP), and collision energies (CE) were individually optimised to ensure accuracy and sensitivity. Data acquisition and preliminary analysis were conducted using Analyst software. Quantification of all targeted metabolites was performed with SCIEX OS‐MQ software (version 1.6.1).

#### 16S rRNA gene full‐length absolute quantitative sequencing and bioinformatic analysis

Absolute quantification 16S rRNA gene amplicons was performed by Majorbio Bio‐Pharm Technology Co., Ltd. Bacterial DNA was extracted from mouse faecal samples using the E.Z.N.A.® soil DNA Kit (Omega Bio‐Tek), according to the manufacturer's instructions. The hypervariable regions V1‐V9 of the bacterial 16S rRNA gene were amplified with primer pairs 27F (5'‐AGRGTTYGATYMTGGCTCAG‐3')/1492R (5'‐RGYTACCTTGTTACGACTT‐3') by a T100 Thermal Cycler (BIO‐RAD). Barcode primers compatible with the PacBio system were used to distinguish individual samples. Purified amplicons were pooled in equimolar amounts and used to construct DNA library with the SMRTbell Prep Kit 3.0 (Pacific Biosciences), following the manufacturer's instructions. Sequencing was performed on the Pacbio Sequel IIe platform (Pacific Biosciences). Raw PacBio reads were processed using the SMRT Link software (version 11.0) to generate high‐quality high‐fidelity (HiFi) reads ≥ 3 full passes and 99% sequence accuracy. HiFi reads were multiplexed and filtered for length, retaining sequences between 1000 and 1800 bp. Operational taxonomic units (OTUs) were clustered using UPARSE (version 11.0) at a 97% sequence similarity threshold. The most abundant sequence for each OTU was selected as a representative sequence. The most abundant sequence with each OTU was selected as the representative, and taxonomic classification was performed using the RDP Classifier (version 2.2). Differential abundance analysis among groups was conducted using the Kruskal–Wallis rank sum test.

### Cell experiments

#### Human intestinal and colon epithelial cell lines

HIEC‐6 (human intestinal epithelial cell line) cells were obtained from QuiCell (QuiCell‐H046, QuiCell Biotechnology Co., Ltd) and cultured in OptiMEM reduced serum medium supplemented with N‐(2‐hydroxyethyl)piperazine‐N'−2‐ethanesulfonic acid (HEPES), epidermal growth factor, and foetal bovine serum (FBS). NCM‐460 (human colon epithelial cell line) cells were obtained from QuiCell (QuiCell‐N502, QuiCell Biotechnology Co., Ltd) and cultured in DMEM basic medium with FBS.

#### Cell viability assessed by cell counting kit‐8 (CCK‐8) assay

To assess cell viability, the culture medium was replaced with fresh medium containing 10% CCK‐8 reagent. Cells were incubated at 37°C for 2–3 h, and absorbance was measured at 450 nm using a microplate reader. Cell viability was calculated using the following formula: cell viability (%) = (absorbance of experimental wells − absorbance of blank wells) ×100%/(absorbance of control wells − absorbance of blank wells).

#### Scratch healing experiments

Scratch healing assays were performed to evaluate the wound healing capacity of intestinal epithelial cells. Cells were seeded in two‐well culture inserts placed in 35 mm μ‐dishes (IBIDI GMBH, Cat: 81176). Once the cell monolayer reached 100% confluence, the inserts were removed using a sterile tweezer, creating a uniform 500 μm cell‐free gap. Following wound creation, the respective treatment interventions were applied to the cells, and the dishes were incubated at 37°C for 12–24 h. The scratch healing rate was then calculated to assess repair capacity.

#### Quantitative polymerase chain reaction (qPCR) of cell tight junction genes

Total RNA was extracted from cells and animal tissues according to the FastPure Cell/Tissue Total RNA Isolation Kit V2 instructions (Vazyme, Cat: RC112‐01). cDNA was synthesised using an Evo M‐MLVRT Premix Kit for qPCR (Accurate Biotechnology (Hunan) Co., Ltd, Cat: AG11706). The primer sequences were as follows: human *TJP1* (F: TGTTCCGTGTTGTGGATACCT; R: GGATGATGCCTCGTTCTACCT), human *GAPDH* (F: CCTCTGACTTCAACAGCGACA; R: ATGAGCTTGACAAAGTGGTCGT), mouse *Tjp1* (F: ACCTCCATAGTGATTTCTGATGT; R: AACTCGGTCATTTTCCTGTAG), and mouse *Gapdh* (F: GACACTGAGCAAGAGAGGCCCTA; R: TGGGATGGAAATTGTGAGGGA). Gene expression levels in all experimental groups were normalised to the endogenous reference gene (*GAPDH or Gapdh*) and relative expression was calculated by comparing each group to the control group.

#### Western blot of cell tight junction proteins

Proteins were extracted from cells and tissues using RIPA Lysis Buffer (Epizyme, Cat: PC102) supplemented with a Protease Inhibitor Cocktail (Epizyme, Cat: GRF101) and a Phosphatase Inhibitor Cocktail (Epizyme, Cat: GRF102). Protein concentrations were determined using the Omni‐Easy^TM^ Ready‐to‐use BCA Protein Assay Kit (Epizyme, Cat: ZJ103). Western blot analysis was performed using the following primary antibodies: anti‐ZO1 tight junction antibody (Abcam, Cat: ab96587), anti‐Occludin antibody (Abcam, Cat: ab216327), and anti‐GAPDH monoclonal antibody (Servicebio, Cat: GB12002‐100). GAPDH was used as the internal loading control.

### Bacterial experiment

#### Bacterial strain

*H. biformis* was purchased from the DSMZ ‐ German Collection of Microorganisms and Cell Cultures GmbH (Type strain NO. 3989), and was cultured in CMC medium in an anaerobic chamber (gas atmosphere N_2_/CO_2_/H_2_, 80:15:5). The supernatant and precipitate of *H. biformis* were derived from cultures of the strain in the conditions described above.

#### Bacterial growth curve detection

To assess the effect of l‐lysine on *H. biformis*, l‐lysine (HY‐N0469, MedChemExpress) was added to cultures at final concentrations of 0, 0.7, 1.4, and 2.8 mM. Co‐cultures were incubated under anaerobic conditions, and bacterial growth was monitored by measuring optical density at 600 nm (OD_600_) at 0, 1.5, 3, 5, 8, 10, 13, and 15 h. Growth curve analysis indicated that 1.4 mM l‐lysine significantly inhibited *H. biformis* growth at 8 h, and was therefore selected as the optimal concentration for subsequent experiments. Supernatant and precipitate samples from *H. biformis* culture with or without 1.4 mM l‐lysine were collected after 8‐h time point for metabolomics and prokaryotic RNA sequencing analyses.

#### Untargeted metabolomic analysis of the precipitate and supernatant of *H. biformis*

Untargeted metabolomic profiling of the *H. biformis* precipitate and supernatant was conducted using LC/GC‐MS. For LC‐MS analysis, pre‐cooled methanol‐water (V: V = 4:1, 4 μg/mL) was added to the samples, followed by the addition of 200 μL of chloroform. Samples were subjected to ultrasonication in an ice water bath and incubated overnight at −40°C. After centrifugation (12,000 rpm, 10 min, 4°C), 400 μL of the supernatant was transferred into a vial for evaporation. The dried residue was reconstituted in 200 μL of methanol‐water (V: V = 1:4), vortexed for 30 s, sonicated in an ice water bath for 3 min in an ice water bath, and incubated at −40°C for 2 h. Then, the mixture was centrifuged for 10 min, and the supernatant was filtered through a 0.22 μm organic phase filter, transferred to an injection vial, and stored at −80°C before LC‐MS analysis.

For GC‐MS analysis, 80 μL of methoxyamine hydrochloride in pyridine (15 mg/mL) was added to each dried samples and incubated at 37°C for 60 min for oximation. Subsequently, 50 μL of BSTFA derivatization reagent, 20 μL of n‐hexane, and 10 μL internal standard mixture were added, and samples were incubated at 70°C for 60 min. Afterward, samples were left at room temperature for 30 min before GC‐MS analysis. Quality control (QC) samples were prepared by pooling equal volumes of all processed samples.

Metabolomic analyses were performed using Waters ACQUITY UPLC I‐Class plus coupled with a Thermo QE ultrahigh performance LC‐tandem high‐resolution mass spectrometer (Waters Corporation), and an Agilent 7890B‐5977B gas chromatography‐mass spectrometer (Agilent Technologies Inc.).

Raw LC‐MS data were processed using Progenesis QI V3.0 software (Nonlinear, Dynamics, Newcastle, UK). GC/MS raw files (.D format) were converted to.abf format using Analysis Base File Converter (version 4.0) and imported into MS‐DIAL (V4.24) for deconvolution and alignment. Compound identification was based on retention time, accurate mass, MS/MS fragmentation patterns, and isotope distribution. Databases used for identification included the Human Metabolome Database (version 2.3), Lipidmaps (version 2.3), METLIN MS/MS Library 2019 software, LuMet Animal 3.0 local database, and LuMet‐GC 5.0 local database.

Processed data matrices were imported into *R* (version 4.3.0, *R* Foundation for statistical computing, Vienna, Austria) for multivariate statistical analysis. PCA, OPLS‐DA, and OPLS‐DA‐Splot were performed. To prevent overfitting, model validity was assessed using seven‐fold cross‐validation and 200‐round response permutation testing. Differential metabolites were identified based on variable importance in projection (VIP > 1.0) from the OPLS‐DA model and statistical significance (*p* < 0.05, two‐tailed Student's *t*‐test). Identified differential metabolites were further subjected to KEGG pathway enrichment analysis (http://www.genome.jp/kegg/) [41].

#### Prokaryotic RNA sequencing and bioinformatic analysis

Total RNA was extracted using the mirVana miRNA Isolation Kit (Ambion), following the manufacturer's protocol. RNA integrity was assessed using the Agilent 2100 Bioanalyser (Agilent Technologies), and only samples with an RNA integrity number (RIN) ≥ 7 were used for subsequent analysis. RNA libraries were constructed with TruSeq Stranded Total RNA with Ribo‐Zero Gold and sequenced on the Illumina HiSeqTM 2500 platform, generating 150 bp/125 bp paired‐end reads.

Raw sequencing data were quality‐filtered using Fastp (version 0.20.1) to obtain high‐quality clean reads [42]. Gene‐level quantification was performed using Rockhooper2 (version 2.0.3), which provided read counts and calculated RPKM values [43]. For differential expression analysis, raw counts were normalised using the EstimateSizeFactors function in the DESeq *R* package (version 1.34.1) [44], and differential genes were identified using the nbinomTest function. Transcripts with a *p*‐value < 0.05 and fold‐change ≥ 2 were considered differentially expressed. KEGG enrichment analyses of the differentially expressed genes were performed using hypergeometric distribution testing to identify significantly affected biological pathways and functions.

#### Statistical analysis

Data were analysed using GraphPad Prism 9.2.0. Quantitative data with normal distribution are presented as the mean ± standard deviation (SD). Student's *t*‐tests were used to compare the means of two samples; one‐way ANOVA was used to compare the means of multiple samples, with post hoc LSD tests for comparisons between the respective groups. For non‐normally distributed continuous variables, the Wilcoxon rank‐sum test was used, with Bonferroni correction applied for post‐hoc comparisons. Repeated measures data were fitted using a general linear model and were analysed with ANOVA. In cases where repeated measures data contained missing values, a mixed‐effects general linear model was used. A *p*‐value < 0.05 was considered statistically significant.

## AUTHOR CONTRIBUTIONS

**Chun‐Hui Jiang**: Writing—original draft; data curation; investigation; formal analysis; visualisation. **Xue Fang**: Funding acquisition; investigation; data curation; formal analysis; writing—review and editing. **Wen Huang**: Funding acquisition; formal analysis. **Ya‐Hui Wang**: Visualisation; formal analysis. **Le Kang**: Formal analysis. **Peng‐Yuan Wang**: Formal analysis. **Chao Xu**: Formal analysis. **Zhao‐Shen Li**: Supervision. **Wen‐Bin Zou**: Conceptualisation; visualisation; supervision; writing—review and editing. **Zhuan Liao**: Supervision; conceptualisation; funding acquisition; project administration.

## CONFLICT OF INTEREST STATEMENT

The authors declare no conflicts of interest.

## ETHICS STATEMENT

This study was approved by the ethics committee of Changhai Hospital, Shanghai, China (GPTAP20231206‐4, CHEC(A.E)2025‐034).

## Supporting information


**Figure S1.** Establishment and characterization of a diarrhea‐predominant IBS (IBS‐D) mouse model.
**Figure S2.** Morphological characterization of *Holdemanella biformis* (*H. biformis*) and evaluation of colonization potential in germ‐free mice.
**Figure S3.** Untargeted metabolome analysis about the precipitate and supernatant of *H. biformis* with or without L‐lysine.
**Figure S4.** Schematic diagram of overall research design.

**Table S1.** Spearman correlations between differential intestinal microbes and metabolites.
**Table S2.** Enrichment pathways of intestinal diseases for six differential metabolites according to the MetaboAnalyst database.
**Table S3.** Screen potential IBS‐related metabolites from six differential metabolites using the GMMAD database.
**Table S4.** Screen potential IBS‐related metabolites from seven differential bacterial genera associated with four metabolites using the GMrepo database.
**Table S5.** The relative abundance of *H. biformis* between individuals with IBS and healthy people pooled in the GMrepo database.
**Table S6.** Intestinal and psychiatric diseases correlated with *H. biformis* were analysed using the GMrepo database.
**Table S7.** Intestinal and psychiatric diseases correlated with L‐lysine were analysed using the GMMAD database.
**Table S8.** KEGG metabolic pathway enrichment of serum neurotransmitters in germ‐free IBS‐D mice.

## Data Availability

All data that support the findings of this study are included in this manuscript and its supplementary information files. The data and scripts for analysis can be found at https://github.com/JiangCH-doctor/IMO-2025-0032. Supplementary materials (figures, tables, graphical abstract, slides, videos, Chinese translated version, and update materials) may be found in the online DOI or iMeta Science http://www.imeta.science/imetaomics/.

## References

[1] Black, Christopher J. , Douglas A. Drossman , Nicholas J. Talley , Johannah Ruddy , and Alexander C. Ford . 2020. “Functional Gastrointestinal Disorders: Advances in Understanding and Management.” The Lancet 396: 1664–1674. 10.1016/s0140-6736(20)32115-2 33049221

[2] Han, Lijuan , Ling Zhao , Yong Zhou , Chao Yang , Teng Xiong , Lin Lu , Yusheng Deng , et al. 2022. “Altered Metabolome and Microbiome Features Provide Clues in Understanding Irritable Bowel Syndrome and Depression Comorbidity.” The International Society for Microbial Ecology Journal 16: 983–996. 10.1038/s41396-021-01123-5 PMC8940891 34750528

[3] Sun, Zheng , Meng Zhang , Min Li , Yogendra Bhaskar , Jinshan Zhao , Youran Ji , Hongbing Cui , et al. 2022. “Interactions between Human Gut Microbiome Dynamics and Sub‐Optimal Health Symptoms During Seafaring Expeditions.” Microbiology Spectrum 10: e0092521. 10.1128/spectrum.00925-21 PMC8754112 35019672

[4] Oldenburg, Marcus , and Hans‐Joachim Jensen . 2019. “Stress and Strain Among Seafarers Related to the Occupational Groups.” International Journal of Environmental Research and Public Health 16: 1153. 10.3390/ijerph16071153 PMC6480598 30935082

[5] Sagaro, Getu Gamo , Marzio Dicanio , Gopi Battineni , Marc Abdul Samad , and Francesco Amenta . 2021. “Incidence of Occupational Injuries and Diseases Among Seafarers: A Descriptive Epidemiological Study Based on Contacts From Onboard Ships to the Italian Telemedical Maritime Assistance Service in Rome, Italy.” BMJ Open 11: e044633. 10.1136/bmjopen-2020-044633 PMC7970292 33727272

[6] Jiang, Chun‐Hui , Xue Fang , Wen Huang , Ji‐Yao Guo , Jia‐Yun Chen , Hong‐Yu Wu , Zhao‐Shen Li , Wen‐Bin Zou , and Zhuan Liao . 2022. “Alterations in the Gut Microbiota and Metabolomics of Seafarers After a Six‐Month Sea Voyage.” Microbiology Spectrum 10: e0189922. 10.1128/spectrum.01899-22 PMC9603232 36197290

[7] Zhang, Yanan , Shuyu Tu , Xingwei Ji , Jianan Wu , Jinxin Meng , Jinsong Gao , Xian Shao , et al. 2024. “Dubosiella Newyorkensis Modulates Immune Tolerance in Colitis via the L‐Lysine‐Activated AhR‐IDO1‐Kyn Pathway.” Nature Communications 15: 1333. 10.1038/s41467-024-45636-x PMC10864277 38351003

[8] Smriga, Miro , and Kunio Torii . 2003. “L‐Lysine Acts Like a Partial Serotonin Receptor 4 Antagonist and Inhibits Serotonin‐Mediated Intestinal Pathologies and Anxiety in Rats.” Proceedings of the National Academy of Sciences 100: 15370–15375. 10.1073/pnas.2436556100 PMC307574 14676321

[9] Smriga, Miro , Makiko Kameishi , Hisayuki Uneyama , and Kunio Torii . 2002. “Dietary L‐Lysine Deficiency Increases Stress‐Induced Anxiety and Fecal Excretion in Rats.” The Journal of Nutrition 132: 3744–3746. 10.1093/jn/132.12.3744 12468617

[10] Schoch, Conrad L. , Stacy Ciufo , Mikhail Domrachev , Carol L. Hotton , Sivakumar Kannan , Rogneda Khovanskaya , and Detlef Leipe , et al. 2020. “NCBI Taxonomy: A Comprehensive Update on Curation, Resources and Tools.” Database (Oxford) 2020: baaa062. 10.1093/database/baaa062 PMC7408187 32761142

[11] Liu, Yuxia , Wenhui Li , Hongxia Yang , Xiaoying Zhang , Wenxiu Wang , Sitong Jia , Beibei Xiang , et al. 2021. “Leveraging 16S rRNA Microbiome Sequencing Data to Identify Bacterial Signatures for Irritable Bowel Syndrome.” Frontiers in Cellular and Infection Microbiology 11: 645951. 10.3389/fcimb.2021.645951 PMC8231010 34178718

[12] El‐Salhy, M . 2023. “Intestinal Bacteria Associated With Irritable Bowel Syndrome and Chronic Fatigue.” Neurogastroenterology & Motility 35: e14621. 10.1111/nmo.14621 37246923

[13] Zhuang, Zhaomeng , Chen Huang , Yiguang Zhang , Bin Lv . 2022. “Effects of Massa Medicata Fermentata on the Intestinal Pathogenic Flagella Bacteria and Visceral Hypersensitivity in Rats With Irritable Bowel Syndrome.” Frontiers in Physiology 13: 1039804. 10.3389/fphys.2022.1039804 PMC9730278 36505059

[14] López‐Gómez, Laura , Ana Bagüés , José Antonio Uranga , and Raquel Abalo . 2020. “Preclinical Models of Irritable Bowel Syndrome [A].” In A Comprehensive Overview of Irritable Bowel Syndrome, edited by Jakub Fichna , 233–276. Academic Press.

[15] Li, Huating , Lei Zhang , Jun Li , Qian Wu , Lingling Qian , Junsheng He , Yueqiong Ni , et al. 2024. “Resistant Starch Intake Facilitates Weight Loss in Humans by Reshaping the Gut Microbiota.” Nature Metabolism 6: 578–597. 10.1038/s42255-024-00988-y PMC10963277 38409604

[16] Qi, Qingqing , Huijie Zhang , Zheyu Jin , Changchun Wang , Mengyu Xia , Bandy Chen , Bomin Lv , et al. 2024. “Hydrogen Sulfide Produced by the Gut Microbiota Impairs Host Metabolism via Reducing GLP‐1 Levels in Male Mice.” Nature Metabolism 6: 1601–1615. 10.1038/s42255-024-01068-x 39030389

[17] Zagato, Elena , Chiara Pozzi , Alice Bertocchi , Tiziana Schioppa , Fabiana Saccheri , Silvia Guglietta , Bruno Fosso , et al. 2020. “Endogenous Murine Microbiota Member Faecalibaculum Rodentium and Its Human Homologue Protect From Intestinal Tumour Growth.” Nature Microbiology 5: 511–524. 10.1038/s41564-019-0649-5 PMC7048616 31988379

[18] Ruiz‐Malagón, Antonio Jesús , María José Rodríguez‐Sanchez , María Jesús Rodríguez‐Sojo , Teresa Vezza , Ivo Pischel , Francesca Algieri , María Elena Rodríguez‐Cabezas , Alba Rodríguez‐Nogales , and Julio Gálvez . 2022. “Intestinal Anti‐Inflammatory and Visceral Analgesic Effects of a Serpylli Herba Extract in an Experimental Model of Irritable Bowel Syndrome in Rats.” Frontiers in Pharmacology 13: 967644. 10.3389/fphar.2022.967644 PMC9479127 36120292

[19] Wang, Fengjun , Brad T. Schwarz , W Vallen Graham , Yingmin Wang , Liping Su , Daniel R. Clayburgh , Clara Abraham , and Jerrold R. Turner . 2006. “IFN‐γ‐Induced TNFR2 Expression Is Required for TNF‐Dependent Intestinal Epithelial Barrier Dysfunction.” Gastroenterology 131: 1153–1163. 10.1053/j.gastro.2006.08.022 PMC1693969 17030185

[20] Tang, Huiling , Qiuping Li , Zhengqi Zha , Yuzhi Jiao , Baowei Yang , Zhaoyan Cheng , Ting Wang , and Hongping Yin . 2024. “Xylan Acetate Ester Ameliorates Ulcerative Colitis through Intestinal Barrier Repair and Inflammation Inhibition via Regulation of Macrophage M1 Polarization.” International Journal of Biological Macromolecules 280: 135551. 10.1016/j.ijbiomac.2024.135551 39276904

[21] Becht, Janine M. , Hendrik Kohlleppel , Roel P. F. Schins , and Angela A. M. Kämpfer . 2024. “Effect of Butyrate on Food‐Grade Titanium Dioxide Toxicity in Different Intestinal In Vitro Models.” Chemical Research in Toxicology 37: 1501–1514. 10.1021/acs.chemrestox.4c00086 PMC11409378 39213652

[22] Chojnacki, Cezary , Aleksandra Błońska , Paulina Konrad , Marcin Chojnacki , Marcin Podogrocki , and Tomasz Poplawski . 2023. “Changes in Tryptophan Metabolism on Serotonin and Kynurenine Pathways in Patients With Irritable Bowel Syndrome.” Nutrients 15: 1262. 10.3390/nu15051262 PMC10005076 36904262

[23] Holtmann, Gerald J. , Alexander C. Ford , and Nicholas J. Talley . 2016. “Pathophysiology of Irritable Bowel Syndrome.” The Lancet Gastroenterology & Hepatology 1: 133–146. 10.1016/s2468-1253(16)30023-1 28404070

[24] Clarke, Gerard , Declan P. McKernan , Gabor Gaszner , Eamonn M. Quigley , John F. Cryan , and Timothy G. Dinan . 2012. “A Distinct Profile of Tryptophan Metabolism Along the Kynurenine Pathway Downstream of Toll‐Like Receptor Activation in Irritable Bowel Syndrome.” Frontiers in Pharmacology 3: 90. 10.3389/fphar.2012.00090 PMC3357104 22661947

[25] Colle, Romain , Perrine Masson , Céline Verstuyft , Bruno Fève , Erwan Werner , Claire Boursier‐Neyret , Bernard Walther , et al. 2020. “Peripheral Tryptophan, Serotonin, Kynurenine, and Their Metabolites in Major Depression: A Case‐Control Study.” Psychiatry and Clinical Neurosciences 74: 112–117. 10.1111/pcn.12944 31599111

[26] Smriga, M. , and K. Torii . 2003. “Metabolic Interactions between Restraint Stress and L‐Lysine: The Effect on Urea Cycle Components.” Amino Acids 24: 435–437. 10.1007/s00726-003-0358-4 12768507

[27] Yao, Shanshan , Huijia Xie , Ya Wang , Nan Shen , Qionglei Chen , Yiting Zhao , Qilu Gu , et al. 2023. “Predictive Microbial Feature Analysis in Patients With Depression After Acute Ischemic Stroke.” Frontiers in Aging Neuroscience 15: 1116065. 10.3389/fnagi.2023.1116065 PMC10076592 37032826

[28] Chen, Yujing , Peilin Meng , Shiqiang Cheng , Yumeng Jia , Yan Wen , Xuena Yang , Yao Yao , et al. 2021. “Assessing the Effect of Interaction Between C‐Reactive Protein and Gut Microbiome on the Risks of Anxiety and Depression.” Molecular Brain 14: 133. 10.1186/s13041-021-00843-1 PMC8418706 34481527

[29] Zhang, Huijie , Li Liu , Shiqiang Cheng , Yumeng Jia , Yan Wen , Xuena Yang , Peilin Meng , et al. 2022. “Assessing the Joint Effects of Brain Aging and Gut Microbiota on the Risks of Psychiatric Disorders.” Brain Imaging And Behavior 16: 1504–1515. 10.1007/s11682-022-00630-z 35076893

[30] Zhang, Qiang , Rong Zou , Min Guo , Mengmeng Duan , Quan Li , and Huajun Zheng . 2021. “Comparison of Gut Microbiota between Adults With Autism Spectrum Disorder and Obese Adults.” PEERJ 9: e10946. 10.7717/peerj.10946 PMC7931713 33717692

[31] Scheepers, Isabella M. , John F. Cryan , Thomaz F. S. Bastiaanssen , Kieran Rea , Gerard Clarke , Heather B. Jaspan , Brian H. Harvey , et al. 2020. “Natural Compulsive‐Like Behaviour In the Deer Mouse (*Peromyscus maniculatus* Bairdii) Is Associated With Altered Gut Microbiota Composition.” European Journal Of Neuroscience 51: 1419–1427. 10.1111/ejn.14610 31663195

[32] Du, Yage , Xiaoying Li , Yu An , Ying Song , and Yanhui Lu . 2022. “Association of Gut Microbiota With Sort‐Chain Fatty Acids and Inflammatory Cytokines in Diabetic Patients With Cognitive Impairment: A Cross‐Sectional, Non‐Controlled Study.” Frontiers in Nutrition 9: 930626. 10.3389/fnut.2022.930626 PMC9355148 35938126

[33] Sanz Herranz, Y. , I. Lopez Almela , E. Gomez Del Pulgar Villanueva , A. Benitez‐Paez , M. Romani Perez , V. Gomez Del Pulgar , H. Y. Sanz , et al. 2020. “New Strain of Holdemanella Biformis for Medicine, and Treating and Preventing Diseases Related to Impaired Glucose Metabolism Selected From Glucose Intolerance and Insulin Resistance.” [Patent]. (WO2020136301‐A1).

[34] Kang, K. , J. Y. Limchaeyeon Meang , S. E. Jo , S. Hee , W. Rhee , and D. H. Lee . 2023. “Crohn's Disease or Ulcerative Colitis.” [Patent].” New Holdemanella Biformis Strain Bbh040 Useful in Pharmaceutical Composition or Health Functional Food for e.g. Preventing or Treating Inflammatory Bowel Diseases, Irritable Bowel Syndrome, Enteritis KR2539776‐B1).

[35] Li, Ting , Ning Ding , Hanqing Guo , Rui Hua , Zehao Lin , Huohuan Tian , Yue Yu , et al. 2024. “A Gut Microbiota‐Bile Acid Axis Promotes Intestinal Homeostasis Upon Aspirin‐Mediated Damage.” Cell Host & Microbe 32: 191–208.e9. 10.1016/j.chom.2023.12.015 PMC10922796 38237593

[36] Wei, Yi‐Han , Xi Ma , Jiang‐Chao Zhao , Xiu‐Qi Wang , and Chun‐Qi Gao . 2023. “Succinate Metabolism and Its Regulation of Host‐Microbe Interactions.” Gut Microbes 15: 2190300. 10.1080/19490976.2023.2190300 PMC10038034 36946592

[37] Yin, Yuhan , Anna Sichler , Josef Ecker , Melanie Laschinger , Gerhard Liebisch , Marcus Höring , Marijana Basic , et al. 2023. “Gut Microbiota Promote Liver Regeneration Through Hepatic Membrane Phospholipid Biosynthesis.” Journal of Hepatology 78: 820–835. 10.1016/j.jhep.2022.12.028 36681162

[38] Hu, Jing Feng , Xin Song , Kan Zhong , Xue Ke Zhao , Fu You Zhou , and Rui Hua Xu , et al. 2023. “Increases Prognostic Value of Clinical‐Pathological Nomogram in Patients With Esophageal Squamous Cell Carcinoma.” Frontiers in Oncology 13: 997776. 10.3389/fonc.2023.997776 PMC9973522 36865805

[39] Paragomi, Pedram , Reza Rahimian , Mohammad Hossein Kazemi , Mohammad Hadi Gharedaghi , Amin Khalifeh‐Soltani , Saeedeh Azary , Abbas Norouzi Javidan , et al. 2014. “Antinociceptive and Antidiarrheal Effects of Pioglitazone in a Rat Model of Diarrhoea‐Predominant Irritable Bowel Syndrome: Role of Nitric Oxide.” Clinical and Experimental Pharmacology and Physiology 41: 118–126. 10.1111/1440-1681.12188 24471407

[40] Pentkowski, Nathan S. , Kimberly K. Rogge‐Obando , Tia N. Donaldson , Samuel J. Bouquin , and Benjamin J. Clark . 2021. “Anxiety and Alzheimer's Disease: Behavioral Analysis and Neural Basis in Rodent Models of Alzheimer's‐Related Neuropathology.” Neuroscience & Biobehavioral Reviews 127: 647–658. 10.1016/j.neubiorev.2021.05.005 PMC8292229 33979573

[41] Kanehisa, Minoru , Miho Furumichi , Yoko Sato , Masayuki Kawashima , and Mari Ishiguro‐Watanabe . 2023. “KEGG for Taxonomy‐Based Analysis of Pathways and Genomes.” Nucleic Acids Research 51: D587–D592. 10.1093/nar/gkac963 PMC9825424 36300620

[42] Bolger, Anthony M. , Marc Lohse , and Bjoern Usadel . 2014. “Trimmomatic: A Flexible Trimmer for Illumina Sequence Data.” Bioinformatics 30: 2114–2120. 10.1093/bioinformatics/btu170 PMC4103590 24695404

[43] Mortazavi, Ali , Brian A. Williams , Kenneth McCue , Lorian Schaeffer , and Barbara Wold . 2008. “Mapping and Quantifying Mammalian Transcriptomes by RNA‐Seq.” Nature Methods 5: 621–628. 10.1038/nmeth.1226 PMC13303166 18516045

[44] Anders, Simon , and Wolfgang Huber . 2010. “Differential Expression Analysis for Sequence Count Data.” Molecular Genome Biology 11: R106. 10.1186/gb-2010-11-10-r106 PMC3218662 20979621

