# Supplementary material for: A reciprocal interaction between L‐lysine and Holdemanella biformis modulates intestinal barrier function and anxiety in irritable bowel syndrome
Source: IMetaOmics. 2025 Aug 6;2(3):e70042. doi: 10.1002/imo2.70042 (PMC12805995; doi:10.1002/imo2.70042)
Supplement: Supplementary file 1 — Figure S1. Establishment and characterization of a diarrhea‐predominant IBS (IBS‐D) mouse model. Figure S2. Morphological characterization of Holdemanella biformis (H. biformis) and evaluation of colonization potential in germ‐free mice. Figure S3. Untargeted metabolome analysis about the precipitate and supernatant of H. biformis with or without L‐lysine. Figure S4. Schematic diagram of overall research design. [file IMO2-2-e70042-s002.docx]

**Supporting information to A reciprocal interaction between L-lysine and *Holdemanella biformis* modulates intestinal barrier function and anxiety in irritable bowel syndrome**

**Running title**: L-lysine - *Holdemanella biformis* interaction alters intestinal barrier and anxiety in IBS

Chun-Hui Jiang^1#^, Xue Fang^1#^, Wen Huang^1#^, Ya-Hui Wang^1#^, Le Kang^1^, Peng-Yuan Wang^1^, Chao Xu^1^, Zhao-Shen Li^1^, Wen-Bin Zou^1*^, Zhuan Liao^1*^

^1^Department of Gastroenterology, Shanghai Key Laboratory of Nautical Medicine and Translation of Drugs and Medical Devices, Changhai Hospital; National Key Laboratory of Immunity and Inflammation, Naval Medical University, Shanghai, 200433, China.

*Correspondence:

[liaozhuan@smmu.edu.cn](mailto:liaozhuan@smmu.edu.cn) (Zhuan Liao), [dr.wenbinzou@hotmail.com](mailto:dr.wenbinzou@hotmail.com) (Wen-Bin Zou)

**Supplementary information**

**Figure S1.** Establishment and characterization of a diarrhea-predominant IBS (IBS-D) mouse model.

**Figure S2.** Morphological characterization of *Holdemanella biformis (H. biformis)* and evaluation of colonization potential in germ-free mice.

**Figure S3.** Untargeted metabolome analysis about the precipitate and supernatant of *H. biformis* with or without L-lysine.

**Figure S4.** Schematic diagram of overall research design.

**Table S1.** Spearman correlations between differential intestinal microbes and metabolites.

**Table S2.** Enrichment pathways of intestinal diseases for six differential metabolites according to the MetaboAnalyst database.

**Table S3.** Screen potential IBS-related metabolites from six differential metabolites using the GMMAD database.

**Table S4.** Screen potential IBS-related metabolites from seven differential bacterial genera associated with four metabolites using the GMrepo database.

**Table S5.** The relative abundance of *H. biformis* between individuals with IBS and healthy people pooled in the GMrepo database.

**Table S6.** Intestinal and psychiatric diseases correlated with *H. biformis* were analysed using the GMrepo database.

**Table S7.** Intestinal and psychiatric diseases correlated with L-lysine were analysed using the GMMAD database.

**Table S8.** KEGG metabolic pathway enrichment of serum neurotransmitters in germ-free IBS-D mice.

**Supplementary Figures**


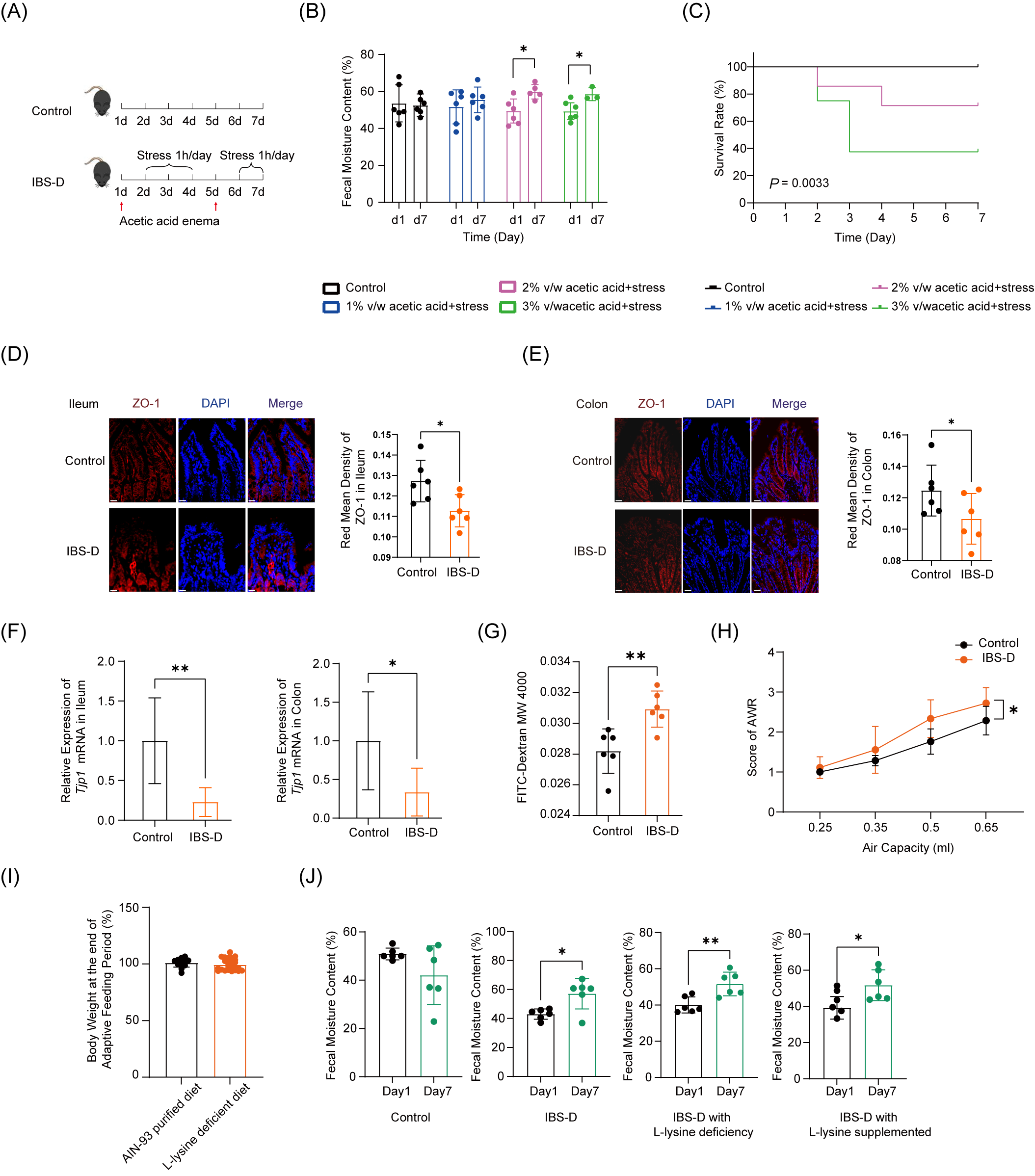


**Figure S1.** Establishment and characterization of a diarrhea-predominant IBS (IBS-D) mouse model. (A) Schematic of model induction protocol comparing IBS-D mouse model construction using three different low-concentration acetic acid enemas (1%, 2%, and 3% v/w) on days 1 and 5, combined with restraint stress for 1 h on days 2-4 and 6-7. (B) Faecal moisture content changes on day 1 and day 7 among IBS-D model mice with three low-concentration acetic acid enemas (1%, 2%, and 3% v/w). (C) Survival rate among IBS-D model mice with three low-concentration acetic acid enemas (1%, 2%, and 3% v/w). (D-E) Representative immunofluorescence images of ZO-1 (left) and red mean density analysis of ZO-1 (right) in the ileum (D) and colon (E) between the control and the IBS-D model mice with 2% v/w acetic acid enemas. Scale bar, 20 μm. (F) qPCR analysis of *Tjp1* mRNA expression in the ileum and colon between the control and the IBS-D model mice with 2% v/w acetic acid enemas (normalized to *Gapdh*). (G) Intestinal permeability assessed by serum FITC-Dextran MW4000 levels between the control and the IBS-D model mice with 2% v/w acetic acid enemas. (H) Visceral hypersensitivity measured by AWR scores to colorectal distension between the control and the IBS-D model mice with 2% v/w acetic acid enemas. (I) Body weight changes at the end of seven-day adaptive feeding period between the groups given AIN-93M purified feed with or without L-lysine. (J) Faecal moisture content changes on day 1 and day 7 among mice in the control group, IBS-D group, IBS-D with L-lysine deficiency group, and IBS-D with L-lysine supplemented group.^*^*p* < 0.05; ^**^*p* < 0.01. IBS-D, diarrhea-predominant irritable bowel disorders; ZO-1: zonula occludens-1; AWR, Abdominal withdrawal reflex.


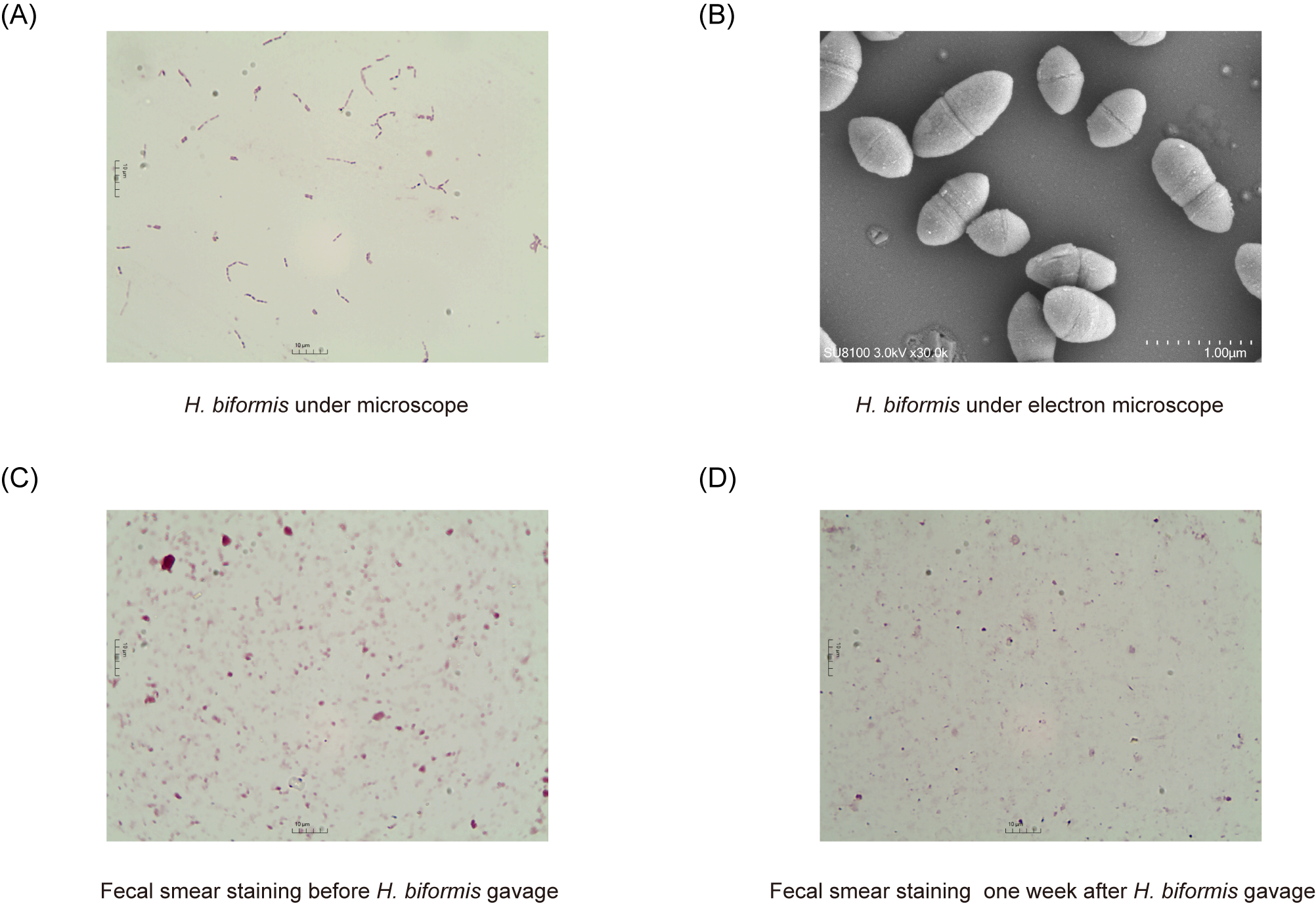


**Figure S2.** Morphological characterization of *Holdemanella biformis* (*H. biformis*) and evaluation of colonization potential in germ-free mice**.** (A) Light microscopy reveals the characteristic coccus-shaped morphology of *H. biformis*. Scale bar, 10 μm. (B) Electron microscopy (×30,000 magnification) of *H. biformis*. Scale bar, 1 μm. (C-D) Gram-stained faecal smears evaluation of *H. biformis* colonization potential in germ-free mice, with (C) showing sterile conditions pre-gavage and (D) absence of *H. biformis* colonization in germ-free mice one week post-gavage (10^8^ CFU). Scale bar, 10 μm.


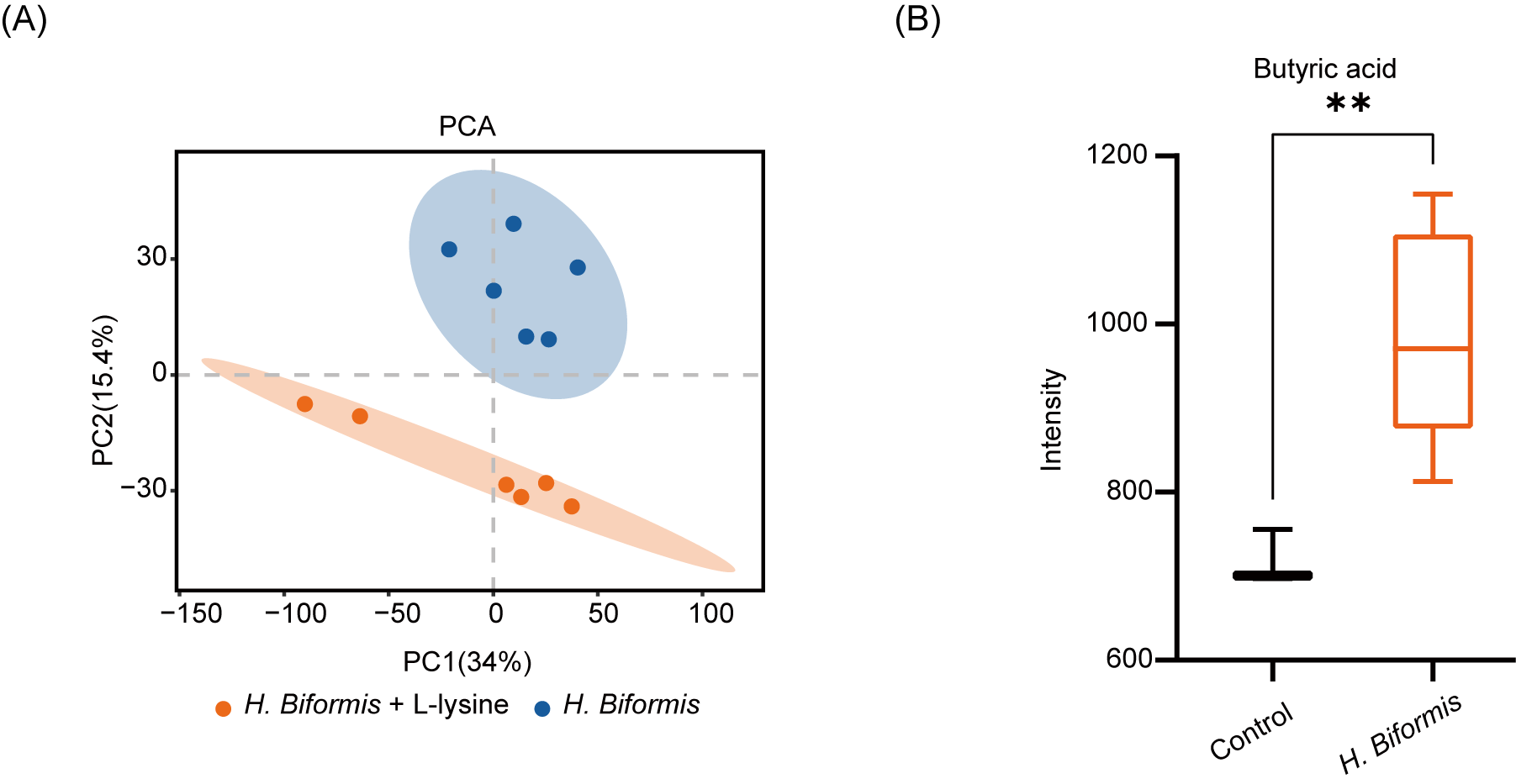


**Figure S3.** Untargeted metabolome analysis about the precipitate and supernatant of *H. biformis* with or without L-lysine. (A) PCA analysis showing metabolic differences between groups. (B) Intensity of butyric acid in the medium containing *H. biformis* compared with control medium by untargeted metabolomics. ^**^*p* < 0.01. PCA, principal component analysis.


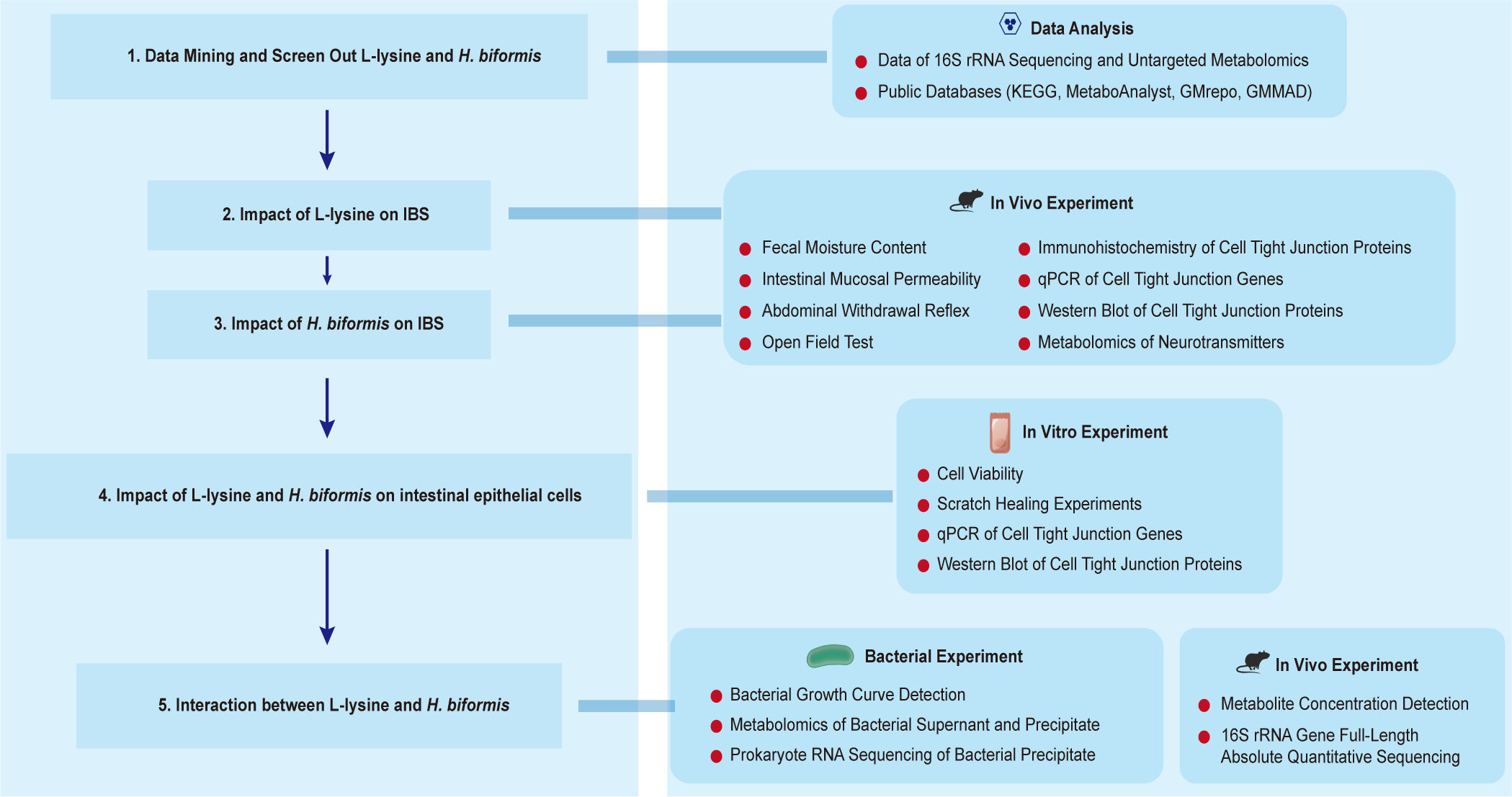


**Figure S4.** Schematic diagram of overall research design.
